# Predicting Prokaryotic Ecological Niches Using Genome Sequence Analysis

**DOI:** 10.1371/journal.pone.0000743

**Published:** 2007-08-15

**Authors:** Garret Suen, Barry S. Goldman, Roy D. Welch

**Affiliations:** 1 Department of Biology, Syracuse University, Syracuse, New York, United States of America; 2 Monsanto, St. Louis, Missouri, United States of America; University College Dublin, Ireland

## Abstract

Automated DNA sequencing technology is so rapid that analysis has become the rate-limiting step. Hundreds of prokaryotic genome sequences are publicly available, with new genomes uploaded at the rate of approximately 20 per month. As a result, this growing body of genome sequences will include microorganisms not previously identified, isolated, or observed. We hypothesize that evolutionary pressure exerted by an ecological niche selects for a similar genetic repertoire in those prokaryotes that occupy the same niche, and that this is due to both vertical and horizontal transmission. To test this, we have developed a novel method to classify prokaryotes, by calculating their Pfam protein domain distributions and clustering them with all other sequenced prokaryotic species. Clusters of organisms are visualized in two dimensions as ‘mountains’ on a topological map. When compared to a phylogenetic map constructed using 16S rRNA, this map more accurately clusters prokaryotes according to functional and environmental attributes. We demonstrate the ability of this map, which we term a “niche map”, to cluster according to ecological niche both quantitatively and qualitatively, and propose that this method be used to associate uncharacterized prokaryotes with their ecological niche as a means of predicting their functional role directly from their genome sequence.

## Introduction

Publicly available sequenced prokaryote genomes will soon number in the thousands. Genomes of the major microbial model organisms have been sequenced, some multiple times; projects to sequence new genomes will select from a pool of increasingly obscure organisms. Meanwhile, meta-genome sequencing projects [1], [2] are attempting to recapitulate whole genome assemblages, resulting in the genomes of microorganisms that have never even been identified or isolated, let alone observed in a laboratory. A great challenge for microbiologists is to exploit this large and expanding amount of sequence data to provide biological information and ecological context for these new genomes [3]–[5]. If a genome sequence is the only thing known about an organism, what can be learned about its specific role and function in an ecosystem? In other words, can we use a genome sequence to identify an organism's ecological niche?

Initial attempts to define the term ‘niche’ focused either on the environment an organism inhabits [6] or the function of an organism [7], but a more contemporary definition incorporates both of these aspects [8]. Every species occupies a niche, but defining the niche of many prokaryote species is difficult because of their surprisingly wide geographical and ecological ranges [9]. The traditional methods used to define the niche of a novel prokaryote are morphological observation, biochemical characterization, and phylogenetic classification by multiple sequence alignment of its 16S rRNA [10]–[12]. If an organism cannot be isolated in the laboratory, however, it cannot be observed morphologically or characterized biochemically. In addition, attempting to identify a niche by phylogenetics alone is proving to be difficult: although there is an apparent connection between phylogeny and niche, phylogenetically distant species sometimes share the same niche, and phylogenetically close species sometimes occupy very different niches. New algorithms that expand phylogenetics to incorporate the entire genome, including those based on average amino acid identity [13], shared gene orthology [14], [15], protein structures or domains [16]–[18], and correlated indel alignments [19], were developed to either verify existing 16S rRNA phylogeny or to suggest new phylogenetic relationships [18]. However, these algorithms are not optimized to discern the genomic relationship between organisms in a comprehensive way, since they ignore or minimize the effects of horizontal gene transfer (HGT).

A genome sequence is the product of adaptation in response to evolutionary pressure. In theory, any set of genes can be transferred between prokaryotes in the same environment [20], [21], and the fixation of new genetic material occurs rapidly relative to eukaryotes, at least in part because of a prokaryote's relatively short generation time. Genetic changes occur through a variety of evolutionary mechanisms, including vertical descent, HGT [20]–[23], duplication and divergence [24], [25], and genome reduction [26], [27]. Although there is disagreement regarding the relative significance of these mechanisms [20], [28], there is no doubt that prokaryotes use all of them to acquire new functionality. In this way, a prokaryotic community can be thought of as a single evolving genomic assemblage, with the environment, rather than the species, defining the organisms that inhabit the assemblage [4].

A prokaryote's genetic repertoire is defined as all of the functionality encoded within its genome [29]. To quantify this, an organism's genome must be broken down into fundamental units, and the fundamental unit of evolution is thought to be the protein domain [30], [31]. A prokaryote's genetic complexity is reflected in the expansion and recombination of proteins, and new proteins are created primarily through the duplication and divergence of protein domains [32]. Therefore, the genetic repertoire of a prokaryote is the distribution of protein domains within its genome, and the genetic repertoires of prokaryotes can be clustered according to the similarity of their protein domain distributions. We hypothesize that these clusters will correspond to specific ecological niches.

In this article, we report on the clustering of over 450 sequenced prokaryotes according to their genetic repertoires. Protein domain distributions were ranked using Spearman's correlation, clustered using multidimensional scaling and force-directed placement [33], and visualized as mountains on a topographical map. Prokaryotes described as occupying the same or similar environment were found within the same mountains on the map, and those sharing similar physiological roles also clustered, thus providing insight as to how these organisms evolved and adapted to their niches. We conclude that this type of DNA sequence metadata analysis can provide useful information about the biology of a prokaryote for which the only available data are its genome sequence and annotation.

## Results

**Figure 1:**
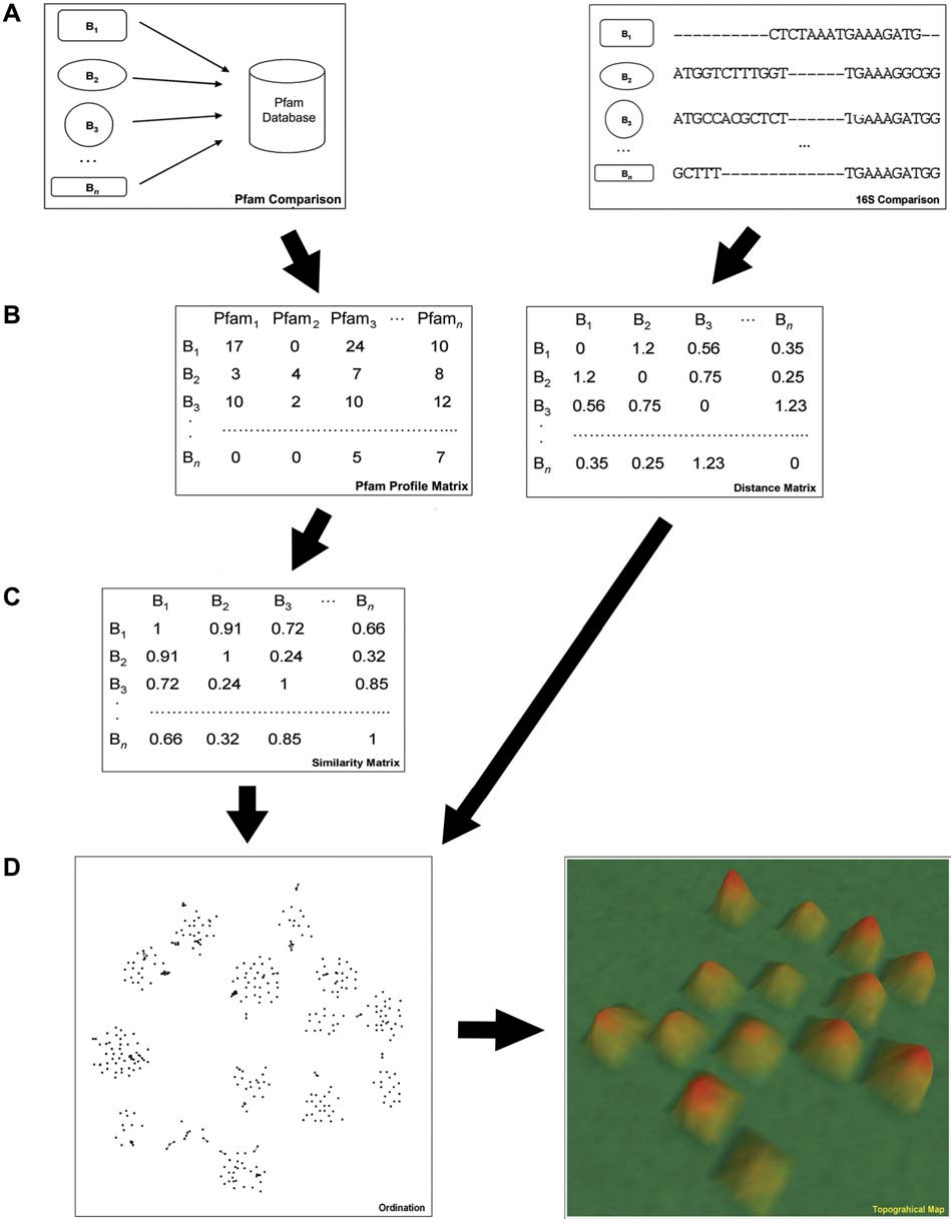
Construction of the niche and phylogenetic maps. The niche map is constructed by comparing all predicted proteins within each prokaryote (B_1_–B_n_) against the Pfam database (a). Likewise, construction of a phylogenetic map is done by performing a multiple sequence alignment using the 16S rRNA sequence from each prokaryote. Each metric is then converted into a Pfam profile and 16S distance matrix, respectively (b). The Pfam profile matrix is further converted into a similarity matrix by applying Spearman's rank correlation (c). Each Prokaryote is then assigned an (x, y) coordinate by applying a combination of multi-dimensional scaling and force-directed placement to both similarity and distance matrices as shown in (d). Finally, a topographical map is generated using the computer program *VxInsight*.

**Figure 2:**
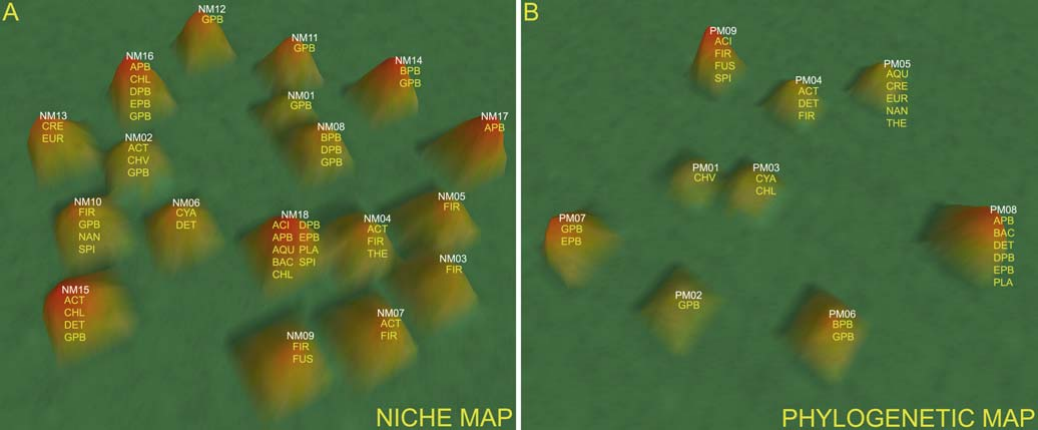
Topographical representation of the niche (a) and phylogenetic (b) maps. Mountain numbers and the corresponding taxonomic groups for each prokaryote within each mountain are shown. Taxonomic abbreviations are as follows: ACI = Acidobacteria; ACT = Actinobacteria; APB = Alphaproteobacteria; AQU = Aquificae; BAC = Bacteroidetes/Chlorobi; BPB = Betaproteobacteria; DPB = Deltaproteobacteria; CHV = Chlamydiae/Verrucomicrobia; CHL = Chloroflexi; CRE = Crenarchaeota; CYA = Cyanobacteria; DET = Deinococcus-Thermus; EPB = Epsilonproteobacteria; EUR = Euryarchaeota; FIR = Firmicutes; FUS = Fusobacteria; GPB = Gammaproteobacteria; NAN = Nanoarchaeota; PLA = Planctomycetes; SPI = Spirochaetes; THE = Thermotogae.

The Protein family (Pfam) annotation was used to measure the protein domain distribution of each genome [34]. The Pfam annotation is an extensive collection of manually curated protein multiple sequence alignments that describes each protein family as a set of conserved domains related to a particular function. We aligned the genetic repertoires, as represented by their predicted proteomes, of over 450 sequenced prokaryotes against the Pfam database to construct a distribution profile for each genome, as shown in Figure 1a, b. This profile is represented as an *n*-component vector of values, with each value corresponding to the total number of instances a particular protein domain occurs within each prokaryote's genome. A map, which we call a “niche map”, was then constructed by clustering each prokaryote's Pfam profile as shown in Figure 1c, d. A phylogenetic map was constructed by the same method using 16S rRNA, so that we could directly compare the niche map to this more traditional analysis, as visualized in the phylogenetic map. A preliminary examination reveals obvious differences with respect to the number of mountains on each map, as shown in Figure 2; the niche and phylogenetic maps contain 18 and 9 mountains, respectively.

Classification of prokaryotic species is based on the phylogenetic distance between each species' 16S rRNA sequence, and the phylogenetic map clusters according to this metric. As there is clearly some overlap between phylogenetic distance and the concept of niche similarity, some mountains on the phylogenetic map will contain organisms that occupy the same niche. When protein domain distribution also correlates with niche similarity, the niche and phylogenetic maps will overlap. However, when 16S rRNA distance and protein domain distribution do not correlate, the niche and phylogenetic maps will diverge and, if genetic repertoire more closely correlates to niche similarity, then divergent mountains on the niche map will correspond to niche similarity, while divergent mountains on the phylogenetic map will correspond to phylogenetic distance.

### Mountains on the Phylogenetic Map Correlate to Phylogenetic Distance

**Figure 3:**
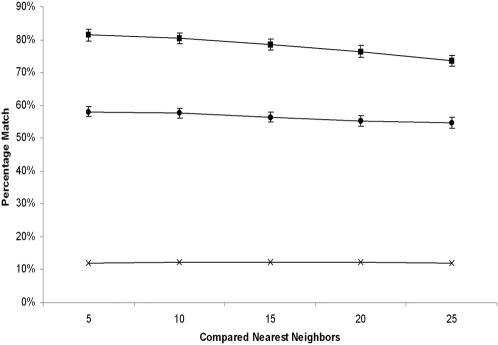
Percentage of comparisons with matches for each prokaryote based on a shared taxonomic group metric. The top 5, 10, 15, 20, and 25 nearest neighbors for each prokaryotic species were retained and their taxonomic groups compared by computing the average number of matches. This analysis was performed for prokaryotes on the niche map (circles) and the phylogenetic map (squares) as shown. In all cases, sequencing bias was taken into account by disregarding all pairs of prokaryotic species that had a Jaccard coefficient greater than 0.90 based on Pfam profile analysis (see Materials and Methods). A control (average of 10 trials) was also performed by randomizing the nearest neighbors for each prokaryotic species (hashes). Error bars for the randomized control are too small to be displayed.

To identify the differences between phylogenetic and niche maps, we assigned phylogenies to all prokaryotic species in both maps, as shown in Figure 2; a tabular representation of this annotation is presented in Table 1, and Table S1. Based on these assignments, 11 out of the 15 taxonomic groups (73%) containing more than one sequenced member had all of their members clustering in a single mountain on the phylogenetic map, as compared to only 5 out of 15 (33%) on the niche map. If clusters are based on phylogenetic distance, then the nearest neighbors of any prokaryote should share in the same phylogenetic group designation; to test this, we applied a shared phylogenetic group metric to the nearest neighbor sets of each prokaryote on both maps. Analysis of both maps across the nearest neighbor sets of 5, 10, 15, 20, and 25 (determined by measuring the Euclidean distance between a prokaryote and all other prokaryotes on the map) indicates that prokaryotes on the phylogenetic map have a higher percentage of nearest neighbors that share the same phylogenetic group than those on the niche map, as shown in Figure 3. Both maps exhibit significant correlation to phylogeny when compared to a randomized control, calculated by randomly assigning nearest neighbors to each prokaryote.

**Table 1:** Composition of mountains for the niche map based on 469 sequenced prokaryotic genomes.

Mount	Species	Represented Genus and Species	Taxonomic Groups
NM01	10	Xanthomonas (G-6), Xylella (G-4)	Gammaproteobacteria (G-10)
NM02	15	Baumannia (G-1), Buchnera (G-1), Chlamydia (V-3), Chlamydophila (V-7), Parachlamydia (V-1), Tropheryma (I-2)	Actinobacteria (I-2), Chlamydiae/Verrucomicrobia (V-11), Gammaproteobacteria (G-2)
NM03	16	Listeria (F-1), Staphylococcus (F-15)	Firmicutes (F)
NM04	19	Alkaliphilus (F-1), Caldicellulosiruptor (F-1), Carboxydothermus (F-1), Clostridium (F-6), Desulfitobacterium (F-2), Desulfotomaculum (F-1), Halothermothrix (F-1), Moorella (F-1), Symbiobacterium (I-1), Syntrophomonas (F-1), Thermoanaerobacter (F-2), Thermotoga (T-1)	Actinobacteria (I-1), Firmicutes (F-17), Thermotogae (T-1)
NM05	19	Bacillus (F-14), Exiguobacterium (F-1), Geobacillus (F-1), Listeria (F-2), Oceanobacillus (F-1)	Firmicutes (F-19)
NM06	21	Anabaena (Y-1), Crocosphaera (Y-1), Cyanobacteria (Y-2),Gloeobacter (Y-1), Nostoc (Y-1), Prochlorococcus (Y-5), Synechococcus (Y-6), Synechocystis (Y-1), Thermosynechococcus (Y-1), Thermus (J-1), Trichodesmium (Y-1)	Cyanobacteria (Y-20), Deinococcus-Thermus (J-1)
NM07	22	Bifidobacterium (I-2), Enterococcus (F-1), Lactobacillus (F-12), Leuconostoc (F-1), Oenococcus (F-1), Pediococcus (F-1), Streptococcus (F-4)	Actinobacteria (I-2), Firmicutes (F-20)
NM08	22	Alkalilimnicola (G-1), Azoarcus (B-1), Bdellovibrio (D-1), Dechloromonas (B-1), Halorhodospira (G-1), Legionella (G-3), Methylobacillus (B-1), Methylococcus (G-1), Neisseria (B-3), Nitrosococcus (G-1), Nitrosomonas (B-2), Nitrosospira (B-1), Psychrobacter (G-3),Thiobacillus (B-1), Thiomicrospira (G-1)	Betaproteobacteria (B-10), Deltaproteobacteria (D-1), Gammaproteobacteria (G-11)
NM09	23	Clostridium (F-3), Enterococcus (F-1), Fusobacterium (K-1), Lactococcus (F-1), Streptococcus (F-17)	Firmicutes (F-22), Fusobacteria (K-1)
NM10	23	Aster yellows witches'-broom (F-1), Borrelia (S-3), Buchnera (G-1), Mesoplasma (F-1), Mycoplasma (F-12), Nanoarchaeum (N-1), Onion yellows (F-1), Treponema (S-2), Ureaplasma (F-1)	Firmicutes (F-16), Gammaproteobacteria (G-1), Nanoarchaeota (N-1), Spirochaetes (S-5)
NM11	27	Alcanivorax (G-1), Chromohalobacter (G-1), Colwellia (G-1), Hahella (G-1), Idiomarina (G-1), Marinobacter (G-1), Photobacterium (G-1), Pseudoalteromonas (G-2), Psychromonas (G-1), Saccharophagus (G-1), Shewanella (G-11), Vibrio (G-5)	Gammaproteobacteria (G-27)
NM12	31	Actinobacillus (G-1), Erwinia (G-1), Escherichia (G-7), Haemophilus (G-1), Mannheimia (G-1), Pasteurella (G-1), Photorhabdus (G-1), Salmonella (G-5), Shigella (G-6), Sodalis (G-1), Yersinia (G-6)	Gammaproteobacteria (G-29)
NM13	31	Aeropyrum (C-1), Archaeoglobus (U-1), Ferroplasma (U-1), Haloarcula (U-1), Halobacterium (U-1), Haloquadratum (U-1), Methanobacterium (U-1), Methanococcoides (U-1), Methanococcus (U-2), Methanoculleus (U-1), Methanopyrus (U-1), Methanosarcina (U-3), Methanosphaera (U-1), Methanospirillum (U-1), Methaosaeta (U-1), Natronomonas (U-1), Picrophilus (U-1), Pyrobaculum (C-1), Pyrococcus (U-3), Sulfolobus (C-3), Thermococcus (U-1), Thermofilum (C-1), Thermoplasma (U-2)	Crenarchaeota (C-6), Euryarchaeota (U-25)
NM14	35	Acinetobacter (G-1), Azotobacter (G-1), Bordetella (B-3), Burkholderia (B-11), Chromobacterium (B-1), Cupriavidus (B-1), Polaromonas (B-2), Pseudomonas (G-10), Ralstonia (B-4), Rhodoferax (B-1)	Betaproteobacteria (B-23), Gammaproteobacteria (G-12)
NM15	36	Acidothermus (I-1), Arthrobacter (I-1), Brevibacterium (I-1), Chloroflexus (G-1), Corynebacterium (I-5), Deinococcus (J-2), Frankia (I-3), Kineococcus (I-1), Leifsonia (I-1), Mycobacterium (I-10), Nocardia (I-1), Nocardioides (I-1), Propionibacterium (I-1), Rhodococcus (I-1), Roseiflexus (L-1), Rubrobacter (I-1), Streptomyces (I-2), Thermobifida (I-1), Thermus (J-1)	Actinobacteria (I-31), Chloroflexi (L-1), Deinococcus-Thermus (J-3), Gammaproteobacteria (G-1)
NM16	39	Anaplasma (A-2), Bartonella (A-2), Buchnera (G-1), Campylobacter (E-2), Coxiella (G-1), Dehalococcoides (L-2), Ehrlichia (A-6), Francisella (G-4), Haemophilus (G-3), Helicobacter (E-5), Lawsonia (D-1), Neorickettsia (A-1), Rickettsia (A-5), Wigglesworthia (G-1), Wolbachia (A-2), Zymomonas (A-1)	Alphaproteobacteria (A-19), Chloroflexi (L-2), Deltaproteobacteria (D-1), Epsilonproteobacteria (E-7), Gammaproteobacteria (G-10)
NM17	40	Acidiphilium (A-1), Agrobacterium (A-1), Bradyrhizobium (A-2), Brucella (A-4), Caulobacter (A-1), Erythrobacter (A-1), Gluconobacter (A-1), Granulobacter (A-1), Hyphomonas (A-1), Jannaschia (A-1), Magnetospirillum (A-2), Maricaulis (A-1), Mesorhizobium (A-2), Nitrobacter (A-2), Novosphingobium (A-1), Paracoccus (A-1), Rhizobium (A-2), Rhodobacter (A-3), Rhodopseudomonas (A-5), Rhodospirillum (A-1), Roseobacter (A-1), Silicibacter (A-2), Sinorhizobium (A-1), Sphingopyxis (A-1), Xanthobacter (A-1)	Alphaproteobacteria (A-40)
NM18	40	Acidobacteria (H-1), Anaeromyxobacter (D-1), Aquifex (Q-1), Bacteroides (O-3), Chlorobium (O-6), Cytophaga (O-1), Dehalococcoides (L-1), Desulfotalea (D-1), Desulfovibrio (D-2), Desulfuromonas (D-1), Flavobacterium (O-1), Geobacter (D-3), Leptospira (S-2), Magnetococcus (A-1), Myxococcus (D-1), Pelobacter (D-2), Pelodictyon (O-2), Pirellula (P-1), Porphyromonas (O-1), Prosthecochloris (O-1), Salinibacter (O-1), Solibacter (H-1), Syntrophobacter (D-1), Syntrophus (D-1), Thiomicrospira (E-1), Wolinella (E-1), delta (D-1)	Alphaproteobacteria (A-1), Acidobacteria (H-2), Aquificae (Q-1), Bacteroidetes/Chlorobi (O-16), Chloroflexi (L-1), Deltaproteobacteria (D-14), Epsilonproteobacteria (E-2), Planctomycetes (P-1), Spirochaetes (S-2)

Each mountain on the map was numbered in ascending order according to the number of prokaryotic species present in each mountain. The represented genus (taxonomic group and number of species in parentheses) and taxonomic groups (abbreviation and total number in parentheses) for each mountain are shown.

The highest percent of shared phylogenetic grouping on the phylogenetic map occurs when the top 5 nearest neighbors are used, producing a value close to 80%, which then steadily decreases to near 70% as the nearest neighbor set increases to 25. It is important to note that 16 out of the 21 represented phylogenetic groups currently have less than 25 sequenced members, so this decrease toward 70% is almost certainly due to the limited number of prokaryotic genomes currently available (Table S2). These results indicate that, as the number of sequenced genomes approaches the set of all prokaryotic species, correlation between the phylogenetic map and phylogeny will approach 100%. In contrast, for the niche map, the percent of shared phylogenetic grouping remains constant at 60% across all nearest neighbor sets, indicating that it will not approach 100% as the set of all sequenced prokaryotic genomes approaches saturation. These data show that both phylogenetic and niche maps correlate to phylogeny, and that the phylogenetic map exhibits better correlation than the niche map.

### Mountains on the Niche Map Correlate to Environment and Function

**Figure 4:**
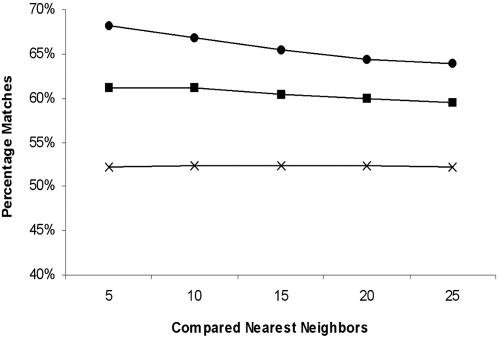
Percentage of comparisons with matches for each prokaryotic species based on an environment and function metric. The top 5, 10, 15, 20, and 25 nearest neighbors for each prokaryotic species were retained and their corresponding environmental and functional data were compared by computing the average number of matches across nine categories. This analysis was performed for prokaryotes on the niche map (circles) and the phylogenetic map (squares) as shown. In all cases, sequencing bias was taken into account by disregarding all pairs of prokaryotic species that had a Jaccard coefficient greater than 0.90 based on Pfam profile analysis (see Materials and Methods). A control (average of 10 trials) was also performed by randomizing the nearest neighbors for each prokaryotic species (hashes). Error bars for all three analyses are too small to be displayed.

To make an objective comparison of phylogenetic and niche maps based on niche similarity, we applied a metric that, although arguably not ideal, is externally derived and relates to the environmental and functional definition of a niche. Specifically, we selected nine categories from the Organism Information dataset associated with the current collection of sequenced prokaryotic genomes (see Materials and Methods for a full list of categories). We then calculated the average percent of categorical matches between each prokaryote and its nearest neighbor sets of 5, 10, 15, 20, and 25 for both phylogenetic and niche maps, as shown in Figure 4. The similarity values for the niche map increase from 65% to 68% as the nearest neighbor sets decrease from 25 to 5, whereas values for the phylogenetic map and randomized control are consistently less than those for the niche map, remaining constant across all nearest neighbor sets, with percentages of 62% and 52%, respectively. This comparison between the phylogenetic and niche maps using these nine Organism Information categories presents the inverse of the phylogeny comparison. These data show that phylogenetic and niche maps both correlate to the Organism Information categories, and that the niche map exhibits better correlation than the phylogenetic map.

### Qualitative Analysis

Phylogeny has a rigorous definition but niche does not. The Organism Information categories used to compare phylogenetic and niche maps relate to niche because they describe aspects of environment and function, but we make no claim that these categories are comprehensive; given the present state of prokaryotic post-genomics, it would be impossible to craft a comprehensive definition of prokaryotic niche that would be universally accepted. It is possible to discuss specific organisms and the niches they inhabit, however. Therefore, to further support the hypothesis that the niche map clusters according to niche similarity, we present a detailed analysis of some mountains on the phylogenetic and niche maps.

### Comparison of Clustering by Phylogeny: the Gammaproteobacteria

The Gammaproteobacteria are a broad group that includes the largest number of sequenced genomes, currently at more than 100, including many from extensively characterized prokaryotes [35]. Members of this group, such as *Escherichia coli, Salmonella typhimurium*, and *Yersinia pestis* are not only prominent model organisms, but also include some of mankind's most pernicious pathogens. An analysis of the phylogenetic map places this diverse group into three mountains (PM02, PM06, and PM07; Table S1). PM02 contains predominantly members of the *Pseudomondales, Legionellales*, and *Thiotrichales*. PM06 contains members of the *Xanthomondales*, including species of *Xanthomonas* and *Xylella*, and members of the *Chromatiales* including *Alkaliminicola* and *Nitrosococcus*. PM07 contains the largest grouping of Gammaproteobacteria, including the *Enterobacteriales*, including *Escherichia* and *Salmonella*, and the *Vibrionales*, including *Vibrio* and *Photobacterium*. Also found in this mountain are the *Pasteurales*, including species of *Mannheimia* and *Haemophilus*, and members of the *Alteromonadales* including *Shewanella* and *Pseudoalteromonas*.

In contrast, the niche map distributes this same set of organisms across nine mountains: NM01, NM02, NM08, NM10, NM11, NM12, NM14, NM15, and NM16 (Table 1). The *Enterobacteriale*s form a tight cluster within NM12 that also includes four members of the *Pasteurales* that are all associated with humans as pathogens. These include the genera *Haemophilus, Mannheinia*, and *Pasteurella*. The only member of the *Enterobacteriales* not found in this mountain is the aphid symbiont *Buchnera*, which is found in NM16 along with a group of Alphaproteobacteria that are either obligate symbionts or pathogens (see below).

The *Xanthomonadales* form a distinct group in NM01 (Table 1) of the plant pathogenic species *Xylella* and *Xanthomonas*. The set of Pfams that distinguish the *Xanthomonadales* from the other groups was analyzed as described in Text S1 and shown in Table S3; many of these proteins degrade carbohydrates, which is consistent with the biology of plant pathogens. In addition, a number of Pfams that correspond to membrane lipoproteins and proteins of uncharacterized function are also found as part of this set. Interestingly, the Gammaproteobacteria *Pseudomonas syringae*, another plant pathogen, ends up in NM14 (Table 1) with the other *Pseudomonadales* and an order of the Betaproteobacteria called the *Burkholderiales*, which contains the genera *Ralstonia, Bordetella*, and *Burkholderia*. What sets this group apart is the predominance of soil and plant associated prokaryotes, many of which are also opportunistic human pathogens (see below).

**Figure 5:**
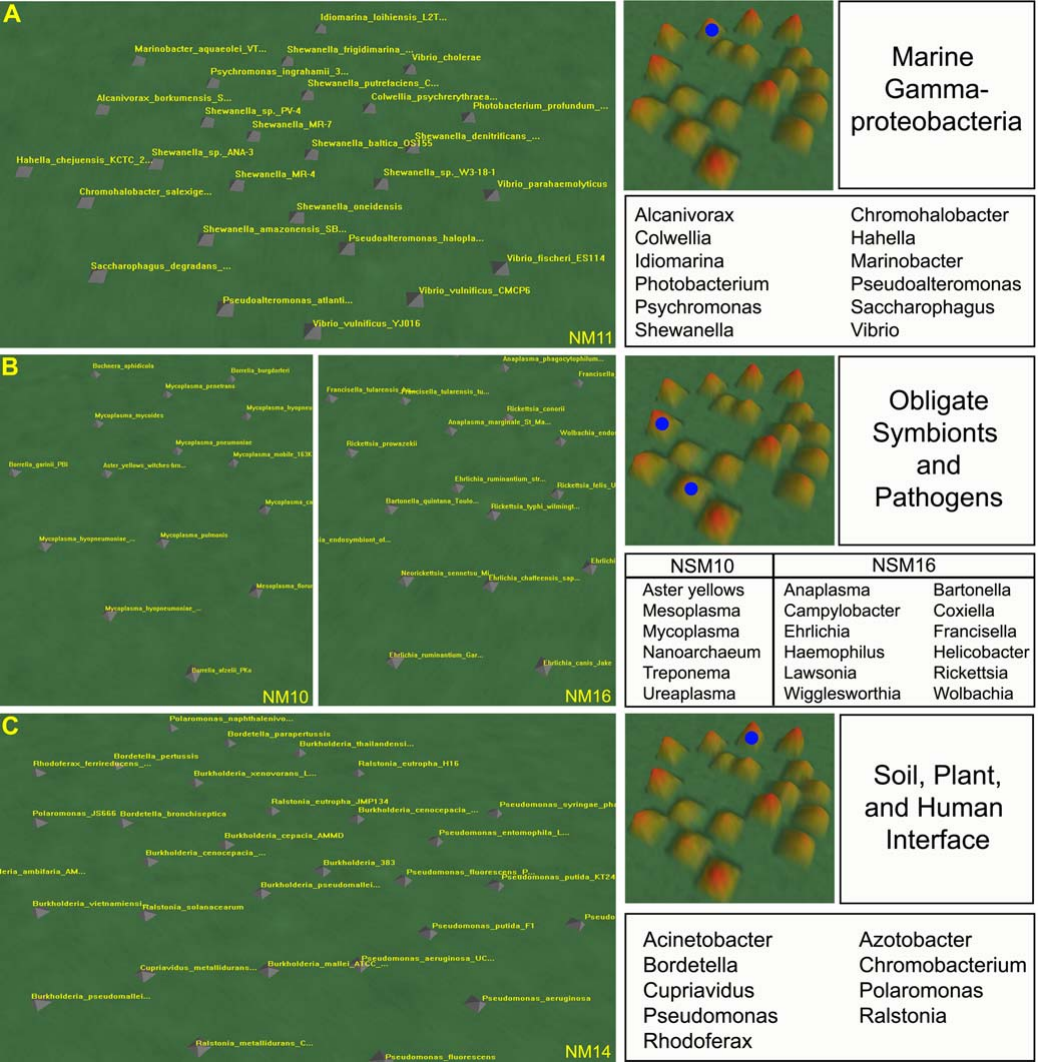
Clustering of prokaryotic species on the niche map. Three groups of prokaryotic species are shown, including the marine Gammaproteobacteria in NM11 that cluster according to phylogeny (a); the obligate symbionts and pathogens in NM10 and NM16 that cluster according to function (b); and the prokaryotes existing at the soil, plant, and human interface in NM14 that cluster according to environment. A high resolution view of each mountain is shown, in addition to the complete niche map labeled with the corresponding mountain (blue circles). The genus of every prokaryote in each mountain is also shown.

Perhaps the group that reveals the most about the ability of the niche map to clustering according to niche is in NM11, (Table 1 and Figure 5a) which contains several different members of the *Alteromonadales, Oceanospirillales*, and the *Vibrionales*. The members of this phylogenetically diverse group within the Gammaproteobacteria are all marine organisms, and the niche map clusters them according to a shared environment. A survey of the Pfams specific to the prokaryotes in NM11 (Table S4) reveal many membrane lipoproteins and proteins of uncharacterized function, similar to those domains observed for the *Xanthomondales*. Interestingly, the marine Gammaproteobacteria in NM11 (Table 1) are found adjacent to the *Xanthomondales* in NM01 (Table 1) on the niche map (see Figure 2).

### Comparison of Clustering by Function: Obligate Symbionts and Pathogens

Obligate symbionts and pathogens describe a diverse group that has undergone massive genome reduction [26], [27], presumably in response to their constrained relationship with a host. Symbionts are characterized as colonizing without necessarily compromising host vigor, whereas pathogens produce a negative impact on the overall health of the host [36]. Organisms that exist as obligate symbionts and pathogens form two tight clusters on the niche map, NM10 and NM16, as shown in Table 1 and Figure 5b.

NM10 (Table 1) consists mainly of obligate pathogens that are distributed across three mountains on the phylogenetic map, PM05, PM07, and PM09 (Table S1) that encompass four different orders including members of the Firmicutes, Gammaproteobacteria, Nanoarchaeota, and Spirochaetes. The majority of prokaryotes found in NM10 (Table 1) belong to the Mollicutes, including all sequenced members of the genera *Mycoplasma*, the human pathogen *Ureaplasma urealyticum*, and the plant pathogens *Aster yellows witches'-broom phytoplasma* and *Onion yellows phytoplasma*. All of these organisms are known to infect a wide variety of hosts, including swine, chickens, plants, and humans. A total of five of the seven sequenced Spirochaetes are also found in this mountain, including all sequenced members of the genera *Borrelia* and *Treponema*. All of these organisms are known to cause a number of diseases in humans, including Lyme disease, Syphilis, and Gingivitis. Interestingly, it has been suggested that the close genomic similarities that exist between the distantly related *Borrelia* and *Mycoplasma* are an indication of convergent evolution [37]. The only sequenced member of the Nanoarchaeota, *Nanoarchaeum equitans*, a symbiont of the archaeon *Ignicoccus*, is also found in this mountain.

The phylogenetic composition of the organisms clustered in NM16 (Table 1) are found distributed across four different mountains on the phylogenetic map, PM02, PM03, PM07, and PM08 (Table S1), and include members of the Alphaproteobacteria, Deltproteobacteria, Epsilonproteobacteria, and Gammaproteobacteria. There is evident sub-clustering within this mountain, with the Epsilonproteobacteria grouping together on the periphery. This sub-cluster includes members of the *Campylobacterales*, including the genera *Helicobacter* and *Campylobacter,* while the rest of this mountain contains the following mixture of Alphaproteobacteria and Gammaproteobacteria: obligate pathogens of the genera *Ehrlichia* that target horses, dogs, and humans; human pathogens of the genera *Rickettsia* and *Neorickettsia*; sheep pathogens of the genera *Anaplasma*; human pathogens of the genera *Francisella*; cattle and human pathogens of the genera *Haemophilus*; human pathogens of the genera *Bartonella*; and the human pathogen *Coxiella brunetii*. Interestingly, the only sequenced pathogenic Deltaproteobacteria, *Lawsonia intracellularis*, a swine pathogen, is also peripherally found in NM16. In addition to these pathogens, three obligate insect symbionts, the Alphaproteobacterium *Wolbachia* and the Gammaproteobacteria *Buchnera* and *Wigglesworthia,* are also found in this mountain. A functional characteristic that distinguishes the organisms in NM16 from those in NM10 (Table 1) is that the majority of prokaryotes in NM10 directly infect humans and plants, while those in NM16 are transmitted to their final host through insect carriers.

Genome reduction in obligate symbionts and parasites might be at least partially responsible for the organisms in NM10 and NM16 (Table 1) being separate from the rest of the niche map, but it does not explain why these organisms form two separate mountains. We compared the Pfam sets between these two mountains, and compiled a list of Pfams specific to each mountain (Table S5 and S6). An analysis of the Pfam set specific to NM10 reveals a majority of ribosomal subunit proteins, whereas the same analysis for NM16 reveals a set that is largely involved in DNA replication, transcription and translation, and cell division. Perhaps the set of Pfams that distinguish NM10 are a hallmark of direct human-related pathogenicity, while the set of Pfams that distinguish NM16 reflect the selective pressures that result from being transmitted via insects.

### Comparison of Clustering by Environment: The Soil, Plant, and Human Interface

Within the niche map, NM14 (Table 1) exemplifies the wide ecological range exhibited by some prokaryotes [9]. The prokaryotes that cluster within NM14 belong to the Betaproteobacteria and the Gammaproteobacteria, and are found distributed across two mountains on the phylogenetic map (PM02 and PM06; Table S1) according to phylogeny. The reason these organisms form a single mountain on the niche map may be due to their common environment, since the organisms in NM14 exist in soils, as plant-associated microbes, as pathogens in humans, or at the interface between these three niches. Soil-specific microbes within this mountain include the nitrogen fixer *Azotobacter vinelandii,* heavy-metal degraders *P. putida* and *Cupriavidus metallidurans*, and members of the genera *Polaromonas, Rhodoferax*, and *Ralstonia*. Exclusive plant-associated microbes include the plant pathogen *P. syringae*, as well as the plant symbiont *B. thailandiensis*. Human pathogens within NM14 include almost all represented members of the genera *Burkholderia*, all members of the genera *Bordetella*, and *P. aeruginosa PA01*. Some of the prokaryotes that also cluster in this mountain exist in more than one environment, such as the human and plant pathogen *P. aeruginosa PA14*; the soil-dwelling and plant associated *P. fluorescens*; the soil-dwelling and human pathogens *Acinetobacter sp. ADP1* and *Chromobacterium violaceum*; and *B. xenovorans*, which is found to exist in all three environments.

Furthermore, NM14 (Table 1) exhibits clustering of prokaryotes that share both niche and morphology. Members of the *Pseudomondales* and *Burkholderiales* are known to thrive in the lungs of Cystic Fibrosis patients, and historically, the *Burkholderiales* were originally classified as belonging to the *Pseudomonadales* based on their morphological similarity. Subsequent studies using 16S rRNA revealed that these prokaryotes belong to different clades altogether [38]. Interestingly, these prokaryotes form mixed biofilms [39] and *P. aeruginosa* is known to promote *B. cepacia* pathogenesis by upregulating certain virulence factors [40]. Therefore, the similarity in morphology shared by both groups and their ability to cooperate in mixed biofilms may suggest that these prokaryotes share a more complex evolutionary history than that proposed by 16S analysis.

An analysis of the Pfams that distinguish this group from other groups on the map reveal that many of these are membrane lipoproteins and proteins of uncharacterized function as shown in Table S7. Interestingly, these Pfams are also characteristic of the *Xanthomondales* and the marine Gammaproteobacteria in NM01 and NM11 (Table 1), respectively. Accordingly, the soil, plant, and human interface prokaryotes found in NM14 are also found adjacent to both the *Xanthomondales* and marine Gammaproteobacteria on the niche map (Figure 2). Differences between Pfam sets could indicate proteins that are specific to a particular niche, such as the carbohydrate degradation enzymes specific to the *Xanthomondales*.

## Discussion

This is the first demonstration of correlation between an organism's genomic repertoire and its ecological niche. We present a novel computational method for clustering organisms according to their protein domain distribution and demonstrate, both quantitatively and qualitatively, that the resulting niche map correlates to the concept of ecological niche [8] better than a phylogenetic map. The accuracy of the niche map will likely improve as new sequenced genomes are added. For example, an earlier iteration of the niche map based on 340 genomes clustered the *Xanthomondales* (NM01, Table 1) and the marine Gammaproteobacteria (NM11, Table 1) into a single mountain (data not shown). The subsequent division into two mountains on the current map resolves these plant pathogens from the marine-associated Gammaproteobacteria.

Through a comparison of phylogenetic and niche maps, we demonstrate that the phylogenetic map exhibits closer correlation to phylogeny than the niche map. This result is expected because, like a phylogenetic tree, the topology of the phylogenetic map is based on a multiple sequence alignment of different organisms' 16S rRNA. The 16S subunit is chosen because it is considered to be both ubiquitous and essential, and is therefore likely to be evolving predominantly through vertical descent [11]. Although there are examples where 16S rRNA is thought to be transmitted through means other than vertical descent [41], the phylogenetic map effectively isolates the outcome of vertical decent from other evolutionary processes, such as HGT, duplication and divergence, and genome reduction. The topology of the phylogenetic map is, therefore, a visualization of one evolutionary mechanism.

We also demonstrate that the niche map exhibits closer correlation than the phylogenetic map to nine Organism Information categories that relate to the definition of niche. The topology of the niche map is based on the clustering of multiple prokaryotes' protein domain distribution, with each distribution representing one prokaryote's genomic repertoire reduced to its fundamental functional elements. In this way, a protein domain distribution is the opposite of a carefully selected sequence; it is the Pfam parts list for a genome and, as such, is a blunt expression of an organism's overall evolutionary outcome at the genome scale. The topology of the niche map is a visualization of all evolutionary mechanisms including, but not limited to vertical decent. If phylogenetic map topology is determined by a subset of the evolutionary mechanisms that determine niche map topology, then those mountains exhibiting the highest degree of overlap between phylogenetic and niche maps represent clusters of organisms whose ecological niche constrains evolution to vertical decent. An example of this phenomenon is the Archaea, the majority of which are extremophiles that have limited interaction with other organisms due to their restrictive environment. The Archaea cluster within a single mountain on both phylogenetic (PM05, Table S1) and niche (NM13, Table 1) maps, except for the symbiont *N. equitans,* which appropriately clusters with other symbionts and pathogens on the niche map (NM10, Table 1).

We report several other specific examples of similarities and differences between phylogenetic and niche maps, using detailed qualitative analysis to demonstrate a correlation between defining characteristics of niche (i.e. environment and function) and the clustering of organisms on the niche map. Although this analysis requires that the organisms be morphologically or physiologically characterized, construction of the niche map does not. Placement of each organism on the niche map is based on an algorithm that incorporates only genome sequence data, so that entirely novel and uncharacterized organisms can be placed on the map as soon as they are sequenced. The purely computational implementation of the niche map also permits the assignment of putative characteristics to unknown organisms based on the shared characteristics of well-studied organisms within the same mountain. This type of ‘guilt-by-association’ analysis is frequently employed in functional genomics, where it is used to assign putative functions to genes and interaction partners to proteins [42]. Although the immediate value of this analysis lies in the practical application of the genome metadata set to predict the function of unknown prokaryotes, it is not outside the realm of possibility that similar forms of protein domain clustering could be extended to eukaryotes. For example, epigenomic protein domain clustering could be used to identify differences between individuals within the same species, and expression profile protein domain clustering could be used to identify and characterize different cell types within the same organism.

Because of our vast sequencing infrastructure, we are understanding prokaryotic diversity genome-first. To bring these data into biological focus, we must unify genomic and morphological taxonomy. In addition to building better algorithms to support or refute phylogeny based on 16S rRNA, we should apply computational rigor to other theoretical concepts of genomic evolution, and then process the vast post-genomic dataset to determine what ideas can withstand scrutiny. If a protein domain represents the smallest functional unit of evolution, and if the role of an organism within its ecological niche can be represented by the sum of its functional parts, then a comparison of a genome's ‘parts lists’ should group organism by ecological niche. Setting all ambiguities regarding the definition of prokaryotic species and niche aside, the approach presented in this report is effective.

## Materials and Methods

### Datasets

In this study, 469 prokaryotic genome sequences were used in the construction of the maps. A total of 381 completed sequences were obtained from the National Institute of Biotechnological Information (NCBI, http://www.ncbi.nlm.nih.gov/genomes/lproks.cgi, accessed: 10/20/2006) and an additional 88 draft genomes were obtained from the Integrated Microbial Genomes database [43] (http://img.jgi.doe.gov/cgi-bin/pub/main.cgi, accessed: 10/20/2006). Sequences of the 16S small ribosomal subunit for the 385 NCBI genomes were obtained from NCBI or the Ribosomal Database Project [44] (http://rdp.cme.msu.edu/, accessed: 10/20/2006) and used in the construction of the phylogenetic map. The Pfam database used in this study was obtained from NCBI's Conserved Domain Database for protein classification [45] (ftp://ftp.ncbi.nih.gov/pub/mmdb/cdd/, accessed: 10/20/2006). Phylogenetic group, environmental, and functional annotations for all prokaryotes used in this study were obtained from the organism information page associated with the microbial genome projects available from NCBI (http://www.ncbi.nlm.nih.gov/genomes/lproks.cgi, accessed: 10/20/2006).

### Construction of the niche map

The predicted proteomes of all 469 prokaryotic genomes was used in the construction of the niche map. Two maps were constructed; one based on the 381 completed prokaryotic genomes from NCBI, and a second map based on all 469 complete and draft genomes. For each map, all proteins were aligned against the Pfam database using RPSBLAST [45], [46], and a Pfam distribution matrix was constructed as shown in Figure 1, with each row representing a different prokaryotic genome and each column specifying a Pfam identification number. Each cell within this matrix was populated with the number of instances each Pfam was found within each genome. A similarity matrix was then constructed by calculating the correlation between each pair of species using Spearman's rank correlation. For each prokaryote, the top 25 prokaryotes that had the highest positive correlation scores were retained, and a combination of multidimensional scaling and force-directed placement was then used to ordinate each prokaryote on a two-dimensional plane. This ordination was done using the *VxOrd* program included as part of the *VxInsight* package [33], with the settings at position 3 on the edge cuts slider and no sub-clustering. These coordinates were then visualized in three-dimensions using the computer program *VxInsight* as shown in Map S1. The resulting topographical niche map clusters prokaryotes with similar Pfam distribution profiles into mountains, with the height of each mountain corresponding to the density of prokaryotes found in that area. Each mountain on this map was then numbered in ascending order according to the number of prokaryotes present in each mountain, as shown in Table 1.

### Construction of the phylogenetic map

A map based on the 16S small ribosomal subunit was created for the 381 completed genome sequences obtained from NCBI. Multiple sequence alignment of all 16S rRNA was done using MUSCLE [47], as shown in Figure 1, and a distance matrix was then computed using the DNADIST program included in the PHYLIP computer package [48]. The Jukes-Cantor evolutionary model was used in the calculation of this distance matrix. For each prokaryote, the top 50 species with the closest distance scores were retained, and a combination of multidimensional scaling and force-directed placement was used to ordinate each genome on a two-dimensional plane. This ordination was done using the *VxOrd* program included as part of the *VxInsight* package [33], with the settings at position 3 on the edge cuts slider and no sub-clustering. These coordinates were then visualized in three-dimensions as a topographical phylogenetic map using *VxInsight* as shown in Map S2. Each mountain on this map was then numbered in ascending order according to the number of prokaryotes present in each mountain, as shown in Table S1. All analysis using the Jukes-Cantor phylogenetic map was also performed on a second map constructed using the Kimura 2-paramater evolutionary model, and similar results were obtained in each case.

### Comparison of niche and phylogenetic maps

To establish if there is a quantitative difference between the niche similarity and vertical descent derived maps, we compared the nearest neighbor sets for each prokaryote. This was done using the niche and phylogenetic maps based on the 381 completed genome sequences from NCBI. The nearest 5, 10, 15, 20, and 25 neighbor sets were obtained by calculating the Euclidean distance between each prokaryote and every other prokaryote on the map, and corrected for sequencing bias.

### Sequencing Bias Correction

An analysis of the complete genome sequences from NCBI reveals that they are skewed toward particular taxonomic groups as shown in Table S2. This sequencing bias is due to a number of factors, including the limitation of technology to sequence only those prokaryotes that can be cultured, and the fact that prokaryotes are usually sequenced based on their interest for research purposes [5], [49]. As a result, there is an overrepresentation of some prokaryotes, and in particular, specific strains of the same species. The clustering of specific strains of the same prokaryote using Pfam may introduce “false positives” because these prokaryotes are likely clustering due to the similar Pfam distributions generated from their nearly identical genomes. To correct for this, we compared the Pfam distributions of all prokaryotes, based on the presence or absence of individual Pfam identification numbers in their respective genomes, and calculated the Jaccard coefficient of similarity (defined as: A∩B/A∪B for two sets A and B). We retained only those pairs of prokaryotes that had a Jaccard coefficient greater than 0.90 (data not shown). All subsequent comparisons between the niche similarity and vertical descent derived maps were done by disregarding any pair of prokaryotes that appeared on this sequencing bias correction list.

### Assessment of clustering using a phylogenetic group metric

To determine the extent at which each map clustered prokaryotes according to phylogenetic groups, we calculated the percentage of nearest neighbors that shared the same phylogenetic group for each prokaryote on the niche and phylogenetic maps. We calculated the 5, 10, 15, 20, and 25 nearest neighbor sets for each species (sequencing bias corrected) and calculated the percentage of nearest neighbors that shared the same phylogenetic group. These percentages were then averaged across all prokaryotes for each nearest neighbor set. A control (average of 10 trials) was also done by randomly assigning nearest neighbors to each prokaryote (sequencing bias corrected) and applying this shared phylogenetic group metric.

### Assessment of clustering according to environment and function

We utilized an objective and externally derived metric that corresponds to both environment and function. We calculated the 5, 10, 15, 20, and 25 nearest neighbor sets for each species (sequencing bias corrected) and counted the number of matches that occurred for a total of 9 Organism Information categories. These categories include *Shape, Arrangement, Endospores, Motility, Oxygen Requirements, Habitat, Temperature,* and *Pathogenicity*. Categories for which each pair of prokaryotes did not both contain data were excluded from this comparison. The percentage of comparisons with matches was averaged across all prokaryotes for each nearest neighbor set. A control (average of 10 trials) was also done by randomly assigning nearest neighbors to each prokaryote (sequencing bias corrected) and applying this metric.

## Supporting Information

Text S1 (0.02 MB DOC)

Table S1 (0.04 MB DOC)

Table S2 (0.04 MB DOC)

Table S3 (0.14 MB DOC)

Table S4 (0.06 MB DOC)

Table S5 (0.04 MB DOC)

Table S6 (0.12 MB DOC)

Table S7 (0.04 MB DOC)

Map S1 (3.90 MB GZ)

Map S2 (3.89 MB GZ)

## References

[1] Riesenfeld CS, Schloss PD, Handelsman J (2004). Metagenomics: genomic analysis of microbial communities.. Annu Rev Genet.

[2] Chen K, Pachter L (2005). Bioinformatics for whole-genome shotgun sequencing of microbial communities.. PLoS Comput Biol.

[3] Cohan FM, Fraser CM, Read T, Nelson KE (2004). Concepts of bacterial biodiversity for the age of genomics.. Microbial Genomes.

[4] Goldenfeld N, Woese C (2007). Biology's next revolution.. Nature.

[5] Martiny JB, Field D (2005). Ecological perspectives on the sequenced genome collection.. Ecology Letters.

[6] Grinell J (1917). The niche-relationships of the California thrasher.. Auk.

[7] Elton C (1927). Animal ecology..

[8] Patten BC, Auble GT (1981). System Theory of the Ecological Niche.. The American Naturalist.

[9] Martiny JB, Bohannan BJ, Brown JH, Colwell RK, Fuhrman JA (2006). Microbial biogeography: putting microorganisms on the map.. Nat Rev Microbiol.

[10] Garrity GM (2001). Bergey's Manual of Systematic Bacteriology..

[11] Woese CR (1987). Bacterial evolution.. Microbiol Rev.

[12] Woese CR, Kandler O, Wheelis ML (1990). Towards a natural system of organisms: proposal for the domains Archaea, Bacteria, and Eucarya.. Proc Natl Acad Sci U S A.

[13] Konstantinidis KT, Tiedje JM (2005). Towards a genome-based taxonomy for prokaryotes.. J Bacteriol.

[14] Ciccarelli FD, Doerks T, von Mering C, Creevey CJ, Snel B (2006). Toward automatic reconstruction of a highly resolved tree of life.. Science.

[15] Snel B, Bork P, Huynen MA (1999). Genome phylogeny based on gene content.. Nat Genet.

[16] Lin J, Gerstein M (2000). Whole-genome trees based on the occurrence of folds and orthologs: implications for comparing genomes on different levels.. Genome Res.

[17] Yang S, Doolittle RF, Bourne PE (2005). Phylogeny determined by protein domain content.. Proc Natl Acad Sci U S A.

[18] Tekaia F, Yeramian E (2005). Genome trees from conservation profiles.. PLoS Comput Biol.

[19] Gupta RS, Griffiths E (2002). Critical issues in bacterial phylogeny.. Theor Popul Biol.

[20] Ochman H, Lerat E, Daubin V (2005). Examining bacterial species under the specter of gene transfer and exchange.. Proc Natl Acad Sci U S A.

[21] Gogarten JP, Doolittle WF, Lawrence JG (2002). Prokaryotic evolution in light of gene transfer.. Mol Biol Evol.

[22] Ochman H, Lawrence JG, Groisman EA (2000). Lateral gene transfer and the nature of bacterial innovation.. Nature.

[23] Boucher Y, Douady CJ, Papke RT, Walsh DA, Boudreau ME (2003). Lateral gene transfer and the origins of prokaryotic groups.. Annu Rev Genet.

[24] Taylor JS, Raes J (2004). Duplication and divergence: the evolution of new genes and old ideas.. Annu Rev Genet.

[25] Ohno S (1970). Evolution by Gene Duplication..

[26] Moran NA (2002). Microbial minimalism: genome reduction in bacterial pathogens.. Cell.

[27] Ochman H, Moran NA (2001). Genes lost and genes found: evolution of bacterial pathogenesis and symbiosis.. Science.

[28] Simonson AB, Servin JA, Skophammer RG, Herbold CW, Rivera MC (2005). Decoding the genomic tree of life.. Proc Natl Acad Sci U S A 102 Suppl.

[29] Lerat E, Daubin V, Ochman H, Moran NA (2005). Evolutionary origins of genomic repertoires in bacteria.. PLoS Biol.

[30] Orengo CA, Thornton JM (2005). Protein families and their evolution-a structural perspective.. Annu Rev Biochem.

[31] Apic G, Gough J, Teichmann SA (2001). Domain combinations in archaeal, eubacterial and eukaryotic proteomes.. J Mol Biol.

[32] Chothia C, Gough J, Vogel C, Teichmann SA (2003). Evolution of the protein repertoire.. Science.

[33] Davidson G, Wylie B, Boyack K (2001). Cluster stability and the use of noise in interpretation of clustering; 2001..

[34] Finn RD, Mistry J, Schuster-Bockler B, Griffiths-Jones S, Hollich V (2006). Pfam: clans, web tools and services.. Nucleic Acids Res.

[35] Lerat E, Daubin V, Moran NA (2003). From gene trees to organismal phylogeny in prokaryotes: the case of the gamma-Proteobacteria.. PLoS Biol.

[36] Dale C, Moran NA (2006). Molecular interactions between bacterial symbionts and their hosts.. Cell.

[37] Fraser CM, Casjens S, Huang WM, Sutton GG, Clayton R (1997). Genomic sequence of a Lyme disease spirochaete, Borrelia burgdorferi.. Nature.

[38] Yabuuchi E, Kosako Y, Oyaizu H, Yano I, Hotta H (1992). Proposal of Burkholderia gen. nov. and transfer of seven species of the genus Pseudomonas homology group II to the new genus, with the type species Burkholderia cepacia (Palleroni and Holmes 1981) comb. nov.. Microbiol Immunol.

[39] Riedel K, Hentzer M, Geisenberger O, Huber B, Steidle A (2001). N-acylhomoserine-lactone-mediated communication between Pseudomonas aeruginosa and Burkholderia cepacia in mixed biofilms.. Microbiology.

[40] Harrison F (2007). Microbial ecology of the cystic fibrosis lung.. Microbiology.

[41] Badger JH, Eisen JA, Ward NL (2005). Genomic analysis of Hyphomonas neptunium contradicts 16S rRNA gene-based phylogenetic analysis: implications for the taxonomy of the orders ‘Rhodobacterales’ and Caulobacterales.. Int J Syst Evol Microbiol.

[42] Oliver S (2000). Guilt-by-association goes global.. Nature.

[43] Markowitz VM, Korzeniewski F, Palaniappan K, Szeto E, Werner G (2006). The integrated microbial genomes (IMG) system.. Nucleic Acids Res.

[44] Cole JR, Chai B, Farris RJ, Wang Q, Kulam-Syed-Mohideen AS (2007). The ribosomal database project (RDP-II): introducing myRDP space and quality controlled public data.. Nucleic Acids Res.

[45] Marchler-Bauer A, Anderson JB, Derbyshire MK, DeWeese-Scott C, Gonzales NR (2007). CDD: a conserved domain database for interactive domain family analysis.. Nucleic Acids Res.

[46] Altschul SF, Madden TL, Schaffer AA, Zhang J, Zhang Z (1997). Gapped BLAST and PSI-BLAST: a new generation of protein database search programs.. Nucleic Acids Res.

[47] Edgar RC (2004). MUSCLE: multiple sequence alignment with high accuracy and high throughput.. Nucleic Acids Res.

[48] Felenstein J (2006). PHYLIP (Phylogeny Inference Package), version 3.6..

[49] Hugenholtz P (2002). Exploring prokaryotic diversity in the genomic era.. Genome Biol.

