# Supplementary material for: Predicting Prokaryotic Ecological Niches Using Genome Sequence Analysis
Source: PLoS One. 2007 Aug 15;2(8):e743. doi: 10.1371/journal.pone.0000743 (PMC1937020; doi:10.1371/journal.pone.0000743)
Supplement: Text S1 — (0.02 MB DOC) [file pone.0000743.s001.doc]

**Calculation of Distinguishing Protein Sets**

To determine the sets of proteins that distinguish the *Xanthomondales* in mountain 2 on the niche similarity map, we calculated the complete set of Pfams that were found in the genomes of all members of this mountain. The set of Pfams that were found to exist in all 10 *Xanthomondales* in this mountain were then retained, and further filtered by removing all Pfams that were found in greater than two-thirds of the remaining organisms on the niche similarity map. It is likely that this set of Pfams is “unique” to the *Xanthomondales* and would result in their clustering on the niche similarity map. All Pfams unique to the *Xanthomondales* are shown in Table S3. This was also done for the marine Gammaproteobacteria in mountain 11 and the prokaryotes at the soil, plant, and human interface in mountain 14, as shown in Table S4 and S7.

To calculate the set of Pfams unique to the obligate symbionts and pathogens found in mountains 10 and 15 on the niche similarity map, we calculated the complete set of Pfams that were found in the genomes of all members within these mountains. Since the genomes of the obligate symbionts and pathogens are small, relative to the other prokaryotes on the map, we found that their Pfam sets are subsets of the Pfam sets in other organisms; in other words, there does not exist a set of Pfams that are found predominantly within the obligate symbionts and pathogens. Therefore, it is likely that each groups’ existing Pfam set, and the small size of this set results in their clustering on the map. We filtered each groups’ complete Pfam set by retaining only those Pfams that were found in all prokaryotes within the group. To further determine the differences that exist between these two groups of obligate symbiont and pathogens, we compared both Pfam sets and retained only those Pfams that were specific to each group, as shown in Table S5 and S6.
