# Supplementary material for: Predicting Prokaryotic Ecological Niches Using Genome Sequence Analysis
Source: PLoS One. 2007 Aug 15;2(8):e743. doi: 10.1371/journal.pone.0000743 (PMC1937020; doi:10.1371/journal.pone.0000743)
Supplement: Table S1 — (0.04 MB DOC) [file pone.0000743.s002.doc]

**Table S1**: Composition of mountains for the vertical descent derived map constructed using the Jukes-Cantor evolutionary model based on 385 sequenced prokaryotic genomes. Each mount on the vertical descent derived map is ordered according to the number of prokaryotes present in the mountain. The represented genus (taxonomic group and number of species in parentheses) and taxonomic groups (abbreviation and total number of species in parentheses) for each mount are also shown. Similar results were obtained for a vertical descent derived map constructed using the Kimura 2-parameter evolutionary model (data not shown).

| **Mount** | **Species** | **Represented Genus** | **Taxonomic Group** |
| --- | --- | --- | --- |
| PM01 | 11 | Chlamydia (V-3), Chlamydophila (V-7), Parachlamydia (V-1) | Chlamydiae/  Verrucomicrobia (V-11) |
| PM02 | 17 | Acinetobacter (G-1), Alcanivorax (G-1), Coxiella (G-1), Francisella (G-4), Legionella (G-3), Pseudomonas (G-5), Psychrobacter (G-1), Xanthomonas (G-1) | Gammaproteobacteria (G-17) |
| PM03 | 21 | Anabaena (Y-1), Cyanobacteria (Y-2), Dehalococcoides (L-2), Gloeobacter (Y-1), Nostoc (Y-1), Prochlorococcus (Y-5), Synechococcus (Y-6), Synechocystis (Y-1), Thermosynechococcus (Y-1), Trichodesmium (Y-1) | Chloroflexi (L-2),  Cyanobacteria (Y-19) |
| PM04 | 29 | Bifidobacterium (I-1), Carboxydothermus (F-1),  Corynebacterium (I-5), Frankia (I-2), Leifsonia (I-1),  Moorella (F-1), Mycobacterium (I-6), Nocardia (I-1), Propionibacterium (I-1), Rhodococcus (I-1), Rubrobacter (I-1), Streptomyces (I-2), Symbiobacterium (I-1), Thermobifida (I-1), Thermus (J-2), Tropheryma (I-2) | Actinobacteria (I-25), Deinococcus-Thermus (J-2), Firmicutes (F-2) |
| PM05 | 30 | Aeropyrum (C-1), Aquifex (Q-1), Archaeoglobus (U-1), Haloarcula (U-1), Halobacterium (U-1), Haloquadratum (U-1), Methanobacterium (U-1), Methanococcoides (U-1), Methanococcus (U-2), Methanopyrus (U-1),  Methanosarcina (U-3), Methanosphaera (U-1),  Methanospirillum (U-1), Nanoarchaeum (N-1),  Natronomonas (U-1), Picrophilus (U-1), Pyrobaculum (C-1), Pyrococcus (U-3), Sulfolobus (C-3), Thermococcus (U-1), Thermoplasma (U-2), Thermotoga (T-1) | Aquificae (Q-1),  Crenarchaeota (C-5), Euryarchaeota (U-22), Nanoarchaeota (N-1), Thermotogae (T-1) |
| PM06 | 39 | Alkalilimnicola (G-1), Azoarcus (B-1), Bordetella (B-3), Burkholderia (B-8), Chromobacterium (B-1),  Dechloromonas (B-1), Methylobacillus (B-1),  Methylococcus (G-1), Neisseria (B-3), Nitrosococcus (G-1), Nitrosomonas (B-2), Nitrosospira (B-1), Polaromonas (B-1), Pseudomonas (G-1), Ralstonia (B-4), Rhodoferax (B-1), Thiobacillus (B-1), Xanthomonas (G-5), Xylella (G-2) | Betaproteobacteria (B-28), Gammaproteobacteria (G-11) |
| PM07 | 64 | Baumannia (G-1), Buchnera (G-3), Campylobacter (E-2), Chromohalobacter (G-1), Colwellia (G-1), Erwinia (G-1), Escherichia (G-7), Haemophilus (G-4), Hahella (G-1),  Idiomarina (G-1), Mannheimia (G-1), Pasteurella (G-1), Photobacterium (G-1), Photorhabdus (G-1),  Pseudoalteromonas (G-2), Pseudomonas (G-3),  Psychrobacter (G-1), Saccharophagus (G-1), Salmonella (G-5), Shewanella (G-5), Shigella (G-6), Sodalis (G-1),  Thiomicrospira (G-1), Vibrio (G-5), Wigglesworthia (G-1), Wolinella (E-1), Yersinia (G-6) | Epsilonproteobacteria (E-3), Gammaproteobacteria (G-61) |
| PM08 | 80 | Agrobacterium (A-1), Anaeromyxobacter (D-1),  Anaplasma (A-2), Bacteroides (O-3), Bartonella (A-2), Bdellovibrio (D-1), Bradyrhizobium (A-1), Brucella (A-4), Caulobacter (A-1), Chlorobium (O-2), Cytophaga (O-1), Deinococcus (J-2), Desulfotalea (D-1), Desulfovibrio (D-2), Ehrlichia (A-5), Erythrobacter (A-1), Geobacter (D-2), Gluconobacter (A-1), Granulobacter (A-1), Helicobacter (E-5), Hyphomonas (A-1), Jannaschia (A-1), Lawsonia (D-1), Magnetospirillum (A-1), Maricaulis (A-1), Mesorhizobium (A-2), Myxococcus (D-1), Neorickettsia (A-1), Nitrobacter (A-2), Novosphingobium (A-1), Pelobacter (D-1), Pelodictyon (O-1), Pirellula (P-1), Porphyromonas (O-1), Rhizobium (A-2), Rhodobacter (A-1), Rhodopseudomonas (A-5),  Rhodospirillum (A-1), Rickettsia (A-5), Roseobacter (A-1), Salinibacter (O-1), Silicibacter (A-2), Sinorhizobium (A-1), Sphingopyxis (A-1), Syntrophus (D-1), Thiomicrospira (E-1), Wolbachia (A-2), Zymomonas (A-1) | Alphaproteobacteria (A-51), Bacteroidetes/Chlorobi (O-9), Deinococcus-Thermus (J-2), Deltaproteobacteria (D-11), Epsilonproteobacteria (E-6), Planctomycetes (P-1) |
| PM09 | 90 | Acidobacteria (H-1), Aster yellows witch’s broom (F-1),  Bacillus (F-12), Borrelia (S-3), Clostridium (F-5), Desulfitobacterium (F-1), Enterococcus (F-1),  Fusobacterium (K-1), Geobacillus (F-1), Lactobacillus (F-6), Lactococcus (F-1), Leptospira (S-2), Listeria (F-3),  Mesoplasma (F-1), Mycoplasma (F-12), Oceanobacillus (F-1), Onion yellows (F-1), Staphylococcus (F-13),  Streptococcus (F-19), Syntrophomonas (F-1),  Thermoanaerobacter (F-1), Treponema (S-2), Ureaplasma (F-1) | Acidobacteria (H-1),  Firmicutes (F-81),  Fusobacteria (K-1), Spirochaetes (S-7) |
