# Supplementary material for: Predicting Prokaryotic Ecological Niches Using Genome Sequence Analysis
Source: PLoS One. 2007 Aug 15;2(8):e743. doi: 10.1371/journal.pone.0000743 (PMC1937020; doi:10.1371/journal.pone.0000743)
Supplement: Table S2 — (0.04 MB DOC) [file pone.0000743.s003.doc]

**Table S2:** Sequencing bias within the completed genome sequences of the prokaryotes in NCBI. The values presented in this table are for the 381 prokaryotic genome sequences as of 10/15/2006.

| **Taxonomic Group** | **Sequenced Genomes** |
| --- | --- |
| Acidobacteria | 1 |
| Aquificae | 1 |
| Fusobacteria | 1 |
| Nanoarchaeota | 1 |
| Planctomycetes | 1 |
| Thermotogae | 1 |
| Chloroflexi | 2 |
| Deinococcus-Thermus | 4 |
| Crenarchaeota | 5 |
| Spirochaetes | 7 |
| Bacteroidetes/Chlorobi | 9 |
| Epsilonproteobacteria | 9 |
| Chlamydiae/Verrucomicrobia | 11 |
| Deltaproteobacteria | 11 |
| Cyanobacteria | 19 |
| Euryarchaeota | 22 |
| Actinobacteria | 25 |
| Betaproteobacteria | 28 |
| Alphaproteobacteria | 52 |
| Firmicutes | 83 |
| Gammaproteobacteria | 98 |
