# Supplementary material for: Predicting Prokaryotic Ecological Niches Using Genome Sequence Analysis
Source: PLoS One. 2007 Aug 15;2(8):e743. doi: 10.1371/journal.pone.0000743 (PMC1937020; doi:10.1371/journal.pone.0000743)
Supplement: Table S3 — (0.14 MB DOC) [file pone.0000743.s004.doc]

**Table S3.** Pfams unique to the *Xanthmondales* found in mountain 2 on the niche similarity

map. Each Pfam in this list is found in all prokaryotes within mountain 2 and in only at most 33% of the prokaryotes on the rest of map. Pfam IDs and their associated annotations are shown.

| **Pfam** | **Pfam annotation** |
| --- | --- |
| pfam00032 | Cytochrom_B_C, Cytochrome b(C-terminal)/b6/petD |
| pfam00112 | Peptidase_C1, Papain family cysteine protease |
| pfam00127 | Copper-bind, Copper binding proteins, plastocyanin/azurin family |
| pfam00150 | Cellulase, Cellulase (glycosyl hydrolase family 5) |
| pfam00274 | Glycolytic, Fructose-bisphosphate aldolase class-I |
| pfam00394 | Cu-oxidase, Multicopper oxidase |
| pfam00450 | Peptidase_S10, Serine carboxypeptidase |
| pfam00520 | Ion_trans, Ion transport protein |
| pfam00545 | Ribonuclease, ribonuclease |
| pfam00553 | CBM_2, Cellulose binding domain |
| pfam00577 | Usher, Fimbrial Usher protein |
| pfam00656 | Peptidase_C14, Caspase domain |
| pfam00667 | FAD_binding_1, FAD binding domain |
| pfam00704 | Glyco_hydro_18, Glycosyl hydrolases family 18 |
| pfam00728 | Glyco_hydro_20, Glycosyl hydrolase family 20, catalytic domain |
| pfam00816 | Histone_HNS, H-NS histone family |
| pfam00856 | SET, SET domain |
| pfam00930 | DPPIV_N, Dipeptidyl peptidase IV (DPP IV) N-terminal region |
| pfam01120 | Alpha_L_fucos, Alpha-L-fucosidase |
| pfam01163 | RIO1, RIO1 family |
| pfam01301 | Glyco_hydro_35, Glycosyl hydrolases family 35 |
| pfam01346 | FKBP_N, Domain amino terminal to FKBP-type peptidyl-prolyl isomerase |
| pfam01371 | Trp_repressor, Trp repressor protein |
| pfam01431 | Peptidase_M13, Peptidase family M13 |
| pfam01915 | Glyco_hydro_3_C, Glycosyl hydrolase family 3 C terminal domain |
| pfam01963 | TraB, TraB family |
| pfam02167 | Cytochrom_C1, Cytochrome C1 family |
| pfam02551 | Acyl_CoA_thio, Acyl-CoA thioesterase |
| pfam02557 | VanY, D-alanyl-D-alanine carboxypeptidase |
| pfam02599 | CsrA, Global regulator protein family |
| pfam02636 | DUF185, Uncharacterized ACR, COG1565 |
| pfam02643 | DUF192, Uncharacterized ACR, COG1430 |
| pfam02649 | DUF198, Uncharacterized ACR, COG1469 |
| pfam02659 | DUF204, Domain of unknown function DUF |
| pfam02836 | Glyco_hydro_2_C, Glycosyl hydrolases family 2, TIM barrel domain |
| pfam03079 | ARD, ARD/ARD' family |
| pfam03169 | OPT, OPT oligopeptide transporter protein |
| pfam03544 | TonB, Gram-negative bacterial tonB protein |
| pfam03550 | LolB, Outer membrane lipoprotein LolB |
| pfam03600 | CitMHS, Citrate transporter |
| pfam03626 | COX4_pro, Prokaryotic Cytochrome C oxidase subunit IV |
| pfam03653 | UPF0093, Uncharacterised protein family (UPF0093) |
| pfam03658 | UPF0125, Uncharacterised protein family (UPF0125) |
| pfam03692 | UPF0153, Uncharacterised protein family (UPF0153) |
| pfam03695 | UPF0149, Uncharacterised protein family (UPF0149) |
| pfam03797 | Autotransporter, Autotransporter beta-domain |
| pfam03918 | CcmH, Cytochrome C biogenesis protein |
| pfam03922 | OmpW, OmpW family |
| pfam03932 | CutC, CutC family |
| pfam03934 | GspK, General secretion pathway protein K |
| pfam03958 | Secretin_N, Bacterial type II/III secretion system short domain |
| pfam03971 | IDH, Monomeric isocitrate dehydrogenase |
| pfam04060 | FeS, Putative Fe-S cluster |
| pfam04134 | DUF393, Protein of unknown function, DUF393 |
| pfam04171 | DUF405, Protein of unknown function (DUF405) |
| pfam04235 | DUF418, Protein of unknown function (DUF418) |
| pfam04286 | DUF445, Protein of unknown function (DUF445) |
| pfam04303 | DUF453, Protein of unknown function (DUF453) |
| pfam04329 | DUF470, Family of unknown function (DUF470) |
| pfam04330 | DUF471, Family of unknown function (DUF471) |
| pfam04331 | DUF472, Family of unknown function (DUF472) |
| pfam04332 | DUF475, Protein of unknown function (DUF475) |
| pfam04338 | DUF481, Protein of unknown function, DUF481 |
| pfam04339 | DUF482, Protein of unknown function, DUF482 |
| pfam04340 | DUF484, Protein of unknown function, DUF484 |
| pfam04341 | DUF485, Protein of unknown function, DUF485 |
| pfam04342 | DUF486, Protein of unknown function, DUF486 |
| pfam04348 | LppC, LppC putative lipoprotein |
| pfam04349 | MdoG, Periplasmic glucan biosynthesis protein, MdoG |
| pfam04350 | PilO, Pilus assembly protein, PilO |
| pfam04351 | PilP, Pilus assembly protein, PilP |
| pfam04354 | ZipA_C, ZipA, C-terminal FtsZ-binding domain |
| pfam04356 | DUF489, Protein of unknown function (DUF489) |
| pfam04361 | DUF494, Protein of unknown function (DUF494) |
| pfam04362 | Iron_traffic, Bacterial Fe(2+) trafficking |
| pfam04366 | DUF500, Family of unknown function (DUF500) |
| pfam04367 | DUF502, Protein of unknown function (DUF502) |
| pfam04371 | PAD_porph, Porphyromonas-type peptidyl-arginine deiminase |
| pfam04375 | HemX, HemX |
| pfam04376 | ATE_N, Arginine-tRNA-protein transferase, N terminus |
| pfam04377 | ATE_C, Arginine-tRNA-protein transferase, C terminus |
| pfam04378 | DUF519, Protein of unknown function (DUF519) |
| pfam04379 | DUF525, Protein of unknown function (DUF525) |
| pfam04380 | DUF526, Protein of unknown function (DUF526) |
| pfam04381 | RdgC, Putative exonuclease, RdgC |
| pfam04386 | SspB, Stringent starvation protein B |
| pfam04389 | Peptidase_M28, Peptidase family M28 |
| pfam04390 | RplB, Rare lipoprotein B family |
| pfam04464 | Glyphos_transf, CDP-Glycerol:Poly(glycerophosphate) glycerophosphotransferase |
| pfam04493 | Endonuclease_5, Endonuclease V |
| pfam04519 | DUF583, Protein of unknown function, DUF583 |
| pfam04546 | Sigma70_ner, Sigma-70, non-essential region |
| pfam04751 | DUF615, Protein of unknown function (DUF615) |
| pfam04768 | DUF619, Protein of unknown function (DUF619) |
| pfam04932 | Wzy_C, O-Antigen Polymerase |
| pfam04999 | FtsL, Cell division protein FtsL |
| pfam05016 | Plasmid_stabil, Plasmid stabilisation system protein |
| pfam05088 | Bac_GDH, Bacterial NAD-glutamate dehydrogenase |
| pfam05118 | Asp_Arg_Hydrox, Aspartyl/Asparaginyl beta-hydroxylase |
| pfam05137 | PilN, Fimbrial assembly protein (PilN) |
| pfam05187 | ETF_QO, Electron transfer flavoprotein-ubiquinone oxidoreductase |
| pfam05209 | MinC_N, Septum formation inhibitor MinC, N-terminal domain |
| pfam05275 | CopB, Copper resistance protein B precursor (CopB) |
| pfam05567 | Neisseria_PilC, Neisseria PilC protein |
| pfam05597 | Phasin, Poly(hydroxyalcanoate) granule associated protein (phasin) |
| pfam05649 | Peptidase_M13_N, Peptidase family M13 |
| pfam05960 | DUF885, Bacterial protein of unknown function (DUF885) |
| pfam06057 | VirJ, Bacterial virulence protein (VirJ) |
| pfam06189 | 5-nucleotidase, 5'-nucleotidase |
| pfam06258 | DUF1022, Protein of unknown function (DUF1022) |
| pfam06293 | Kdo, Lipopolysaccharide kinase (Kdo/WaaP) family |
| pfam06481 | COX_ARM, COX Aromatic Rich Motif |
| pfam06509 | HtpX_N, HtpX N-terminus |
| pfam06539 | DUF1112, Protein of unknown function (DUF1112) |
| pfam06835 | DUF1239, Protein of unknown function (DUF1239) |
| pfam06843 | DUF1243, Protein of unknown function (DUF1243) |
| pfam06853 | DUF1249, Protein of unknown function (DUF1249) |
| pfam07055 | scADH, Short-chain alcohol dehydrogenase |
| pfam07209 | DUF1415, Protein of unknown function (DUF1415) |
| pfam07221 | GlcNAc_2-epim, N-acylglucosamine 2-epimerase (GlcNAc 2-epimerase) |
| pfam07238 | PilZ, PilZ domain |
| pfam07396 | Porin_O_P, Phosphate-selective porin O and P |
