# Supplementary material for: Predicting Prokaryotic Ecological Niches Using Genome Sequence Analysis
Source: PLoS One. 2007 Aug 15;2(8):e743. doi: 10.1371/journal.pone.0000743 (PMC1937020; doi:10.1371/journal.pone.0000743)
Supplement: Table S4 — (0.06 MB DOC) [file pone.0000743.s005.doc]

**Table S4.** Pfams unique to the marine Gammaproteobacteria in mountain 11 on the niche similarity map. Each Pfam in this list is found in all prokaryotes within mountain 11 and in only at most 33% of the prokaryotes on the rest of map. Pfam IDs and their associated annotations are shown.

| **Pfam ID** | **Pfam Annotation** |
| --- | --- |
| pfam00325 | Crp, Bacterial regulatory proteins, crp family |
| pfam00520 | Ion_trans, Ion transport protein |
| pfam01142 | TruD, tRNA pseudouridine synthase D (TruD) |
| pfam01346 | FKBP_N, Domain amino terminal to FKBP-type peptidyl-prolyl isomerase |
| pfam02508 | Rnf-Nqr, Rnf-Nqr subunit, membrane protein |
| pfam02551 | Acyl_CoA_thio, Acyl-CoA thioesterase |
| pfam02635 | DrsE, DsrE/DsrF-like family |
| pfam03116 | NQR2_RnfD_RnfE, NQR2, RnfD, RnfE family |
| pfam03550 | LolB, Outer membrane lipoprotein LolB |
| pfam03658 | UPF0125, Uncharacterised protein family (UPF0125) |
| pfam03695 | UPF0149, Uncharacterised protein family (UPF0149) |
| pfam03749 | SfsA, Sugar fermentation stimulation protein |
| pfam03872 | RseA_N, Anti sigma-E protein RseA, N-terminal domain |
| pfam03958 | Secretin_N, Bacterial type II/III secretion system short domain |
| pfam04241 | DUF423, Protein of unknown function (DUF423) |
| pfam04262 | Glu_cys_ligase, Glutamate-cysteine ligase |
| pfam04287 | DUF446, Domain of unknown function, DUF446 |
| pfam04333 | VacJ, VacJ like lipoprotein |
| pfam04348 | LppC, LppC putative lipoprotein |
| pfam04354 | ZipA_C, ZipA, C-terminal FtsZ-binding domain |
| pfam04356 | DUF489, Protein of unknown function (DUF489) |
| pfam04362 | Iron_traffic, Bacterial Fe(2+) trafficking |
| pfam04375 | HemX, HemX |
| pfam04380 | DUF526, Protein of unknown function (DUF526) |
| pfam04381 | RdgC, Putative exonuclease, RdgC |
| pfam04390 | RplB, Rare lipoprotein B family |
| pfam04401 | DUF540, Protein of unknown function (DUF540) |
| pfam04546 | Sigma70_ner, Sigma-70, non-essential region |
| pfam04751 | DUF615, Protein of unknown function (DUF615) |
| pfam04952 | AstE_AspA, Succinylglutamate desuccinylase / Aspartoacylase family |
| pfam04999 | FtsL, Cell division protein FtsL |
| pfam05088 | Bac_GDH, Bacterial NAD-glutamate dehydrogenase |
| pfam05137 | PilN, Fimbrial assembly protein (PilN) |
| pfam05157 | GSPII_E_N, GSPII_E N-terminal domain |
| pfam05166 | DUF709, Family of unknown function (DUF709) |
| pfam05896 | NQRA, Na(+)-translocating NADH-quinone reductase subunit A (NQRA) |
| pfam06175 | MiaE, tRNA-(MS[2]IO[6]A)-hydroxylase (MiaE) |
| pfam06835 | DUF1239, Protein of unknown function (DUF1239) |
| pfam06843 | DUF1243, Protein of unknown function (DUF1243) |
| pfam07023 | DUF1315, Protein of unknown function (DUF1315) |
