# Supplementary material for: Predicting Prokaryotic Ecological Niches Using Genome Sequence Analysis
Source: PLoS One. 2007 Aug 15;2(8):e743. doi: 10.1371/journal.pone.0000743 (PMC1937020; doi:10.1371/journal.pone.0000743)
Supplement: Table S5 — (0.04 MB DOC) [file pone.0000743.s006.doc]

**Table S5.** Pfams unique to the obligate pathogens found in mountain 10 on the niche similarity map. Comparisons were made between the full set of Pfams between the obligate pathogens and symbionts in mountains 10 and 16, and this list represents those Pfams that are found in all prokaryotes in mountain 10 and in none of the prokaryotes in mountain 16. Pfam IDs and their associated annotations are shown.

| **Pfam** | **Pfam Annotation** |
| --- | --- |
| pfam00164 | Ribosomal_S12, Ribosomal protein S12 |
| pfam00177 | Ribosomal_S7, Ribosomal protein S7p/S5e |
| pfam00203 | Ribosomal_S19, Ribosomal protein S19 |
| pfam00238 | Ribosomal_L14, Ribosomal protein L14p/L23e |
| pfam00281 | Ribosomal_L5, Ribosomal protein L5 |
| pfam00297 | Ribosomal_L3, Ribosomal protein L3 |
| pfam00298 | Ribosomal_L11, Ribosomal protein L11, RNA binding domain |
| pfam00312 | Ribosomal_S15, Ribosomal protein S15 |
| pfam00333 | Ribosomal_S5, Ribosomal protein S5, N-terminal domain |
| pfam00338 | Ribosomal_S10, Ribosomal protein S10p/S20e |
| pfam00344 | SecY, eubacterial secY protein |
| pfam00347 | Ribosomal_L6, Ribosomal protein L6 |
| pfam00366 | Ribosomal_S17, Ribosomal protein S17 |
| pfam00410 | Ribosomal_S8, Ribosomal protein S8 |
| pfam00411 | Ribosomal_S11, Ribosomal protein S11 |
| pfam00416 | Ribosomal_S13, Ribosomal protein S13/S18 |
| pfam00466 | Ribosomal_L10, Ribosomal protein L10 |
| pfam00573 | Ribosomal_L4, Ribosomal protein L4/L1 family |
| pfam00590 | TP_methylase, Tetrapyrrole (Corrin/Porphyrin) Methylases |
| pfam00673 | Ribosomal_L5_C, ribosomal L5P family C-terminus |
| pfam00687 | Ribosomal_L1, Ribosomal protein L1p/L10e family |
| pfam00861 | Ribosomal_L18p, Ribosomal L18p/L5e family |
| pfam03167 | UDG, Uracil DNA glycosylase superfamily |
| pfam03719 | Ribosomal_S5_C, Ribosomal protein S5, C-terminal domain |
| pfam03947 | Ribosomal_L2_C, Ribosomal Proteins L2, C-terminal domain |
