# Supplementary material for: Predicting Prokaryotic Ecological Niches Using Genome Sequence Analysis
Source: PLoS One. 2007 Aug 15;2(8):e743. doi: 10.1371/journal.pone.0000743 (PMC1937020; doi:10.1371/journal.pone.0000743)
Supplement: Table S6 — (0.12 MB DOC) [file pone.0000743.s007.doc]

**Table S6.** Pfams unique to the obligate pathogens and symbionts found in mountain 16 on the niche similarity map. Comparisons were made between the full set of Pfams between the obligate pathogens and symbionts in mountains 10 and 16, and this list represents those Pfams that are found in all prokaryotes in mountain 16 and in none of the prokaryotes in mountain 10. Pfam IDs and their associated annotations are shown.

| **Pfam** | **Pfam Annotation** |
| --- | --- |
| pfam00006 | ATP-synt_ab, ATP synthase alpha/beta family, nucleotide-binding domain |
| pfam00012 | HSP70, Hsp70 protein |
| pfam00083 | Sugar_tr, Sugar (and other) transporter |
| pfam00085 | Thioredoxin, Thioredoxin |
| pfam00091 | Tubulin, Tubulin/FtsZ family, GTPase domain |
| pfam00106 | adh_short, short chain dehydrogenase |
| pfam00109 | ketoacyl-synt, Beta-ketoacyl synthase, N-terminal domain |
| pfam00117 | GATase, Glutamine amidotransferase class-I |
| pfam00118 | Cpn60_TCP1, TCP-1/cpn60 chaperonin family |
| pfam00119 | ATP-synt_A, ATP synthase A chain |
| pfam00155 | Aminotran_1_2, Aminotransferase class I and II |
| pfam00166 | Cpn10, Chaperonin 10 Kd subunit |
| pfam00204 | DNA_gyraseB, DNA gyrase B |
| pfam00206 | Lyase_1, Lyase |
| pfam00226 | DnaJ, DnaJ domain |
| pfam00231 | ATP-synt, ATP synthase |
| pfam00266 | Aminotran_5, Aminotransferase class-V |
| pfam00270 | DEAD, DEAD/DEAH box helicase |
| pfam00275 | EPSP_synthase, EPSP synthase (3-phosphoshikimate 1-carboxyvinyltransferase) |
| pfam00289 | CPSase_L_chain, Carbamoyl-phosphate synthase L chain, N-terminal domain |
| pfam00306 | ATP-synt_ab_C, ATP synthase alpha/beta chain, C terminal domain |
| pfam00317 | Ribonuc_red_lgN, Ribonucleotide reductase, all-alpha domain |
| pfam00348 | polyprenyl_synt, Polyprenyl synthetase |
| pfam00383 | dCMP_cyt_deam_1, Cytidine and deoxycytidylate deaminase zinc-binding region |
| pfam00398 | RrnaAD, Ribosomal RNA adenine dimethylase |
| pfam00436 | SSB, Single-strand binding protein family |
| pfam00453 | Ribosomal_L20, Ribosomal protein L20 |
| pfam00472 | RF-1, Peptidyl-tRNA hydrolase domain |
| pfam00521 | DNA_topoisoIV, DNA gyrase/topoisomerase IV, subunit A |
| pfam00551 | Formyl_trans_N, Formyl transferase |
| pfam00557 | Peptidase_M24, metallopeptidase family M24 |
| pfam00575 | S1, S1 RNA binding domain |
| pfam00578 | AhpC-TSA, AhpC/TSA family |
| pfam00580 | UvrD-helicase, UvrD/REP helicase |
| pfam00625 | Guanylate_kin, Guanylate kinase |
| pfam00664 | ABC_membrane, ABC transporter transmembrane region |
| pfam00684 | DnaJ_CXXCXGXG, DnaJ central domain (4 repeats) |
| pfam00692 | dUTPase, dUTPase |
| pfam00696 | AA_kinase, Amino acid kinase family |
| pfam00730 | HhH-GPD, HhH-GPD superfamily base excision DNA repair protein |
| pfam00772 | DnaB, DnaB-like helicase N terminal domain |
| pfam00829 | Ribosomal_L21p, Ribosomal prokaryotic L21 protein |
| pfam00849 | PseudoU_synth_2, RNA pseudouridylate synthase |
| pfam00883 | Peptidase_M17, Cytosol aminopeptidase family, catalytic domain |
| pfam00889 | EF_TS, Elongation factor TS |
| pfam00986 | DNA_gyraseB_C, DNA gyrase B subunit, carboxyl terminus |
| pfam01000 | RNA_pol_A_bac, RNA polymerase Rpb3/RpoA insert domain |
| pfam01025 | GrpE, GrpE |
| pfam01043 | SecA_PP_bind, SecA preprotein cross-linking domain |
| pfam01121 | CoaE, Dephospho-CoA kinase |
| pfam01132 | EFP, Elongation factor P (EF-P) OB domain |
| pfam01138 | RNase_PH, 3' exoribonuclease family, domain 1 |
| pfam01195 | Pept_tRNA_hydro, Peptidyl-tRNA hydrolase |
| pfam01196 | Ribosomal_L17, Ribosomal protein L17 |
| pfam01225 | Mur_ligase, Mur ligase family, catalytic domain |
| pfam01230 | HIT, HIT domain |
| pfam01250 | Ribosomal_S6, Ribosomal protein S6 |
| pfam01252 | Peptidase_A8, Signal peptidase (SPase) II |
| pfam01272 | GreA_GreB, Prokaryotic transcription elongation factor, GreA/GreB, C-terminal domain |
| pfam01300 | Sua5_yciO_yrdC, yrdC domain |
| pfam01327 | Pep_deformylase, Polypeptide deformylase |
| pfam01367 | 5_3_exonuc, 5'-3' exonuclease, C-terminal SAM fold |
| pfam01416 | PseudoU_synth_1, tRNA pseudouridine synthase |
| pfam01434 | Peptidase_M41, Peptidase family M41 |
| pfam01513 | NAD_kinase, ATP-NAD kinase |
| pfam01556 | DnaJ_C, DnaJ C terminal region |
| pfam01715 | IPPT, IPP transferase |
| pfam01743 | PolyA_pol, Poly A polymerase head domain |
| pfam01751 | Toprim, Toprim domain |
| pfam01765 | RRF, Ribosome recycling factor |
| pfam01790 | LGT, Prolipoprotein diacylglyceryl transferase |
| pfam01795 | Methyltransf_5, MraW methylase family |
| pfam01807 | zf-CHC2, CHC2 zinc finger |
| pfam01820 | Dala_Dala_lig_N, D-ala D-ala ligase N-terminus |
| pfam02096 | 60KD_IMP, 60Kd inner membrane protein |
| pfam02130 | UPF0054, Uncharacterized protein family UPF0054 |
| pfam02223 | Thymidylate_kin, Thymidylate kinase |
| pfam02491 | FtsA, Cell division protein FtsA |
| pfam02687 | FtsX, Predicted permease |
| pfam02699 | YajC, Preprotein translocase subunit |
| pfam02739 | 5_3_exonuc_N, 5'-3' exonuclease, N-terminal resolvase-like domain |
| pfam02779 | Transket_pyr, Transketolase, pyrimidine binding domain |
| pfam02780 | Transketolase_C, Transketolase, C-terminal domain |
| pfam02786 | CPSase_L_D2, Carbamoyl-phosphate synthase L chain, ATP binding domain |
| pfam02801 | Ketoacyl-synt_C, Beta-ketoacyl synthase, C-terminal domain |
| pfam02867 | Ribonuc_red_lgC, Ribonucleotide reductase, barrel domain |
| pfam02874 | ATP-synt_ab_N, ATP synthase alpha/beta family, beta-barrel domain |
| pfam02882 | THF_DHG_CYH_C, Tetrahydrofolate dehydrogenase/cyclohydrolase, NAD(P)-binding domain |
| pfam03054 | tRNA_Me_trans, tRNA methyl transferase |
| pfam03099 | BPL_LipA_LipB, Biotin/lipoate A/B protein ligase family |
| pfam03118 | RNA_pol_A_CTD, Bacterial RNA polymerase, alpha chain C terminal domain |
| pfam03120 | DNA_ligase_OB, NAD-dependent DNA ligase OB-fold domain |
| pfam03147 | FDX-ACB, Ferredoxin-fold anticodon binding domain |
| pfam03449 | GreA_GreB_N, Prokaryotic transcription elongation factor, GreA/GreB, N-terminal domain |
| pfam03462 | PCRF, PCRF domain |
| pfam03483 | B3_4, B3/4 domain |
| pfam03485 | Arg_tRNA_synt_N, Arginyl tRNA synthetase N terminal domain |
| pfam03725 | RNase_PH_C, 3' exoribonuclease family, domain 2 |
| pfam03796 | DnaB_C, DnaB-like helicase C terminal domain |
| pfam03989 | DNA_gyraseA_C, DNA gyrase C-terminal domain, beta-propeller |
| pfam04055 | Radical_SAM, Radical SAM superfamily |
| pfam04539 | Sigma70_r3, Sigma-70 region 3 |
| pfam04542 | Sigma70_r2, Sigma-70 region 2 |
| pfam04545 | Sigma70_r4, Sigma-70, region 4 |
| pfam04561 | RNA_pol_Rpb2_2, RNA polymerase Rpb2, domain 2 |
| pfam06418 | CTP_synth_N, CTP synthase N-terminus |
| pfam06421 | LepA_C, GTP-binding protein LepA C-terminus |
| pfam06480 | FtsH_ext, FtsH Extracellular |
| pfam06723 | MreB_Mbl, MreB/Mbl protein |
