# Supplementary material for: Predicting Prokaryotic Ecological Niches Using Genome Sequence Analysis
Source: PLoS One. 2007 Aug 15;2(8):e743. doi: 10.1371/journal.pone.0000743 (PMC1937020; doi:10.1371/journal.pone.0000743)
Supplement: Table S7 — (0.04 MB DOC) [file pone.0000743.s008.doc]

**Table S7.** Pfams unique to prokaryotes found at the Soil, Plant, and Human interface in mountain 14 on the niche similarity map. Each Pfam in this list is found in all prokaryotes within mountain 14 and in only at most 33% of the prokaryotes on the rest of map. Pfam IDs and their associated annotations are shown.

| **Pfam** | **Pfam Annotation** |
| --- | --- |
| pfam03550 | LolB, Outer membrane lipoprotein LolB |
| pfam03922 | OmpW, OmpW family |
| pfam04303 | DUF453, Protein of unknown function (DUF453) |
| pfam04359 | DUF493, Protein of unknown function (DUF493) |
| pfam04376 | ATE_N, Arginine-tRNA-protein transferase, N terminus |
| pfam04377 | ATE_C, Arginine-tRNA-protein transferase, C terminus |
| pfam04386 | SspB, Stringent starvation protein B |
| pfam04390 | RplB, Rare lipoprotein B family |
| pfam04751 | DUF615, Protein of unknown function (DUF615) |
| pfam04999 | FtsL, Cell division protein FtsL |
| pfam05728 | UPF0227, Uncharacterised protein family (UPF0227) |
| pfam06073 | DUF934, Bacterial protein of unknown function (DUF934) |
| pfam06945 | DUF1289, Protein of unknown function (DUF1289) |
| pfam01478 | Peptidase_A24, Type IV leader peptidase family |
| pfam02515 | CoA_transf_3, CoA-transferase family III |
| pfam02625 | XdhC_CoxI, XdhC and CoxI family |
| pfam02705 | K_trans, K+ potassium transporter |
| pfam03653 | UPF0093, Uncharacterised protein family (UPF0093) |
| pfam04279 | IspA, Intracellular septation protein A |
| pfam04333 | VacJ, VacJ like lipoprotein |
| pfam04378 | DUF519, Protein of unknown function (DUF519) |
| pfam04546 | Sigma70_ner, Sigma-70, non-essential region |
| pfam05195 | AMP_N, Aminopeptidase P, N-terminal domain |
| pfam06835 | DUF1239, Protein of unknown function (DUF1239) |
| pfam04355 | SmpA_OmlA, SmpA / OmlA family |
| pfam05494 | Tol_Tol_Ttg2, Toluene tolerance, Ttg2 |
