# Supplementary material for: Predicting Prokaryotic Ecological Niches Using Genome Sequence Analysis
Source: PLoS One. 2007 Aug 15;2(8):e743. doi: 10.1371/journal.pone.0000743 (PMC1937020; doi:10.1371/journal.pone.0000743)
Supplement: Map S1 — (3.90 MB GZ) [file pone.0000743.s009.gz › Map_S1/Software/VxUsersGuide1_1.pdf]

VxInsight®

User's Guide

Version 1.1

VisWave, LLC
12042 Sundial NE
Albuquerque, NM 87122
info@viswave.com
http://www.viswave.com

©VisWave, LLC, 1997-2004

CONTENTS

GETTING STARTED........1
INSTALLATION........1
CONTENTS OF THE VXINSIGHT DIRECTORY........2
RUNNING *VXINSIGHT*........2

GENERAL OVERVIEW........3
THE *VXINSIGHT* WINDOW........3
MAIN MENU........4
SIDE MENU........5
MAIN GRAPHICS WINDOW........6
TERRAIN VIEW MODE........6
WIREFRAME MODE........7
SCATTERPLOT MODE........8
ZOOMING AND ROTATING........9
Zooming........9
Rotating........9
SELECT SINGLE DATA OBJECT........10
SELECT DATA OBJECTS SUBSET (DRAG BOX SELECTION)........10
OBJECT INFORMATION VIEW........11
QUERY MENU........11
Query Box........11
Legend........12
Query Mode........12

AUTOMATED DATA IMPORT........14
VXIMPORT........14

MANUAL DATA IMPORT........20
REQUIRED FILES........20
YOUR DATA SOURCE........21
Microsoft Access........21
Microsoft Excel........21
Converting Excel data to Microsoft Access........22
Converting Text data to Microsoft Access........22
THE VXINSIGHT FILES (DB, CONFIG, STOP)........24
CONSTRUCTION OF A VXINSIGHT SIM FILE (*.SIM)........27
VXORD™: MAPPING YOUR DATA........28
Input files........28
Output files........28
Advanced features........29
CREATING A CACHE FILE........30
Database not found........31

SAMPLE ANALYSIS........32

PLUGINS........33
VXGS (VXINSIGHT AND GENESPRING)........33
Passing data between VxInsight and GeneSpring........34
LOADING YOUR OWN PLUGIN INTO VXINSIGHT........36

SUPPORT AND CONTACTS........38
General Contact Information........38
General Company Information........38

GETTING STARTED

Installation

The VxInsight installation software comes in the form of a self-extracting executable, either on a CDROM, or from an Internet download. Simply double-click on the vx_2xxx.exe executable. This begins the installation process.

A dialog box that looks like this should pop up. Click the "Setup" button and follow the instructions. During the installation you will be required to register your software using a registration dialog box like the one shown.

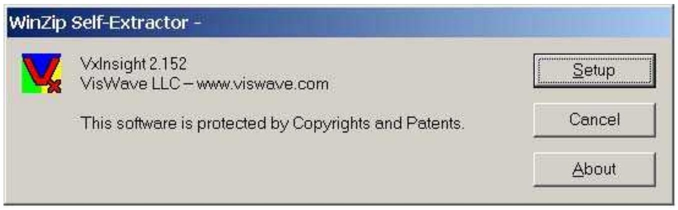

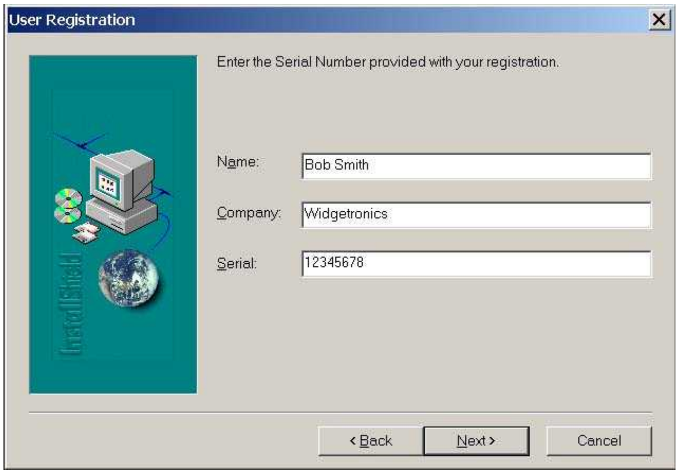

Please enter your name, company name, and the registration number or key you received with your software. You cannot proceed until all fields have been entered. If you do not have your registration number, please contact VisWave support (see Support and Contacts section of this document).

Near the end of the installation you will be asked if you want to install software specific to plugins. If you are going to use any plugins, respond with a yes; if you are not going to use plugins or are not familiar with them, respond no. Plugins can always be installed at a later time.

After installation you will have two icons on your desktop that look like this. Installation of the VxInsight software is now complete.[1]

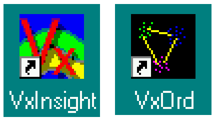

[1] Note that if installing a "plugin" form of VxInsight, such as VxGS, no icons will appear on your desktop. See the plugin section for details.

©VisWave, LLC, 1997-2004

Contents of the VxInsight Directory

The *VxInsight* installation process creates the following files in the folder you choose during the installation:

1. vxinsight.exe (*VxInsight* program)
2. vxord.exe (*VxInsight* ordination or clustering program)
3. vx_icon.ico, vxord.ico (icon files)
4. tcl83.dll, tk83.dll (Tcl/Tk libraries)
5. Uninst.isu
6. DATASETS directory (contains subdirectories with sample datasets)
7. GENERICTCLTK directory
8. IMAGES, TCL, UI_ADAPTER, VX_FILES, VXORD_TCL directories (needed to run *VxInsight*)
9. PLUGINS directory (for use with any plugins)

Running *VxInsight*

To get started:

1. Double-click on the *VxInsight* icon that was installed to your desktop, OR click on the "Start" button on your screen, then on the "Programs" item, "VxInsight" folder, and "VxInsight" item. This will start *VxInsight*. The program takes several seconds to start up and initialize. You will see a splash screen with a rolling and color changing image of VxInsight. The purpose of this splash screen is to determine default graphics settings for VxInsight, based on the graphics capability of your computer. After the VxInsight image goes away, the program is ready for you to proceed.

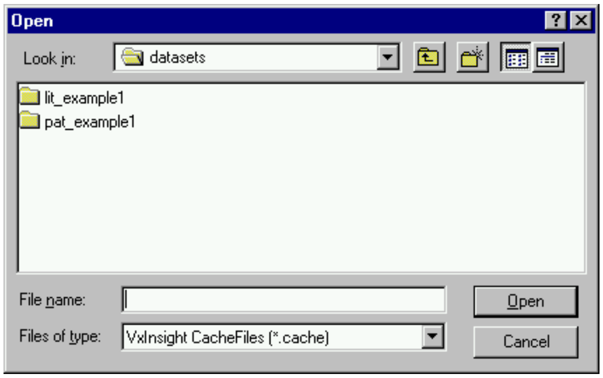

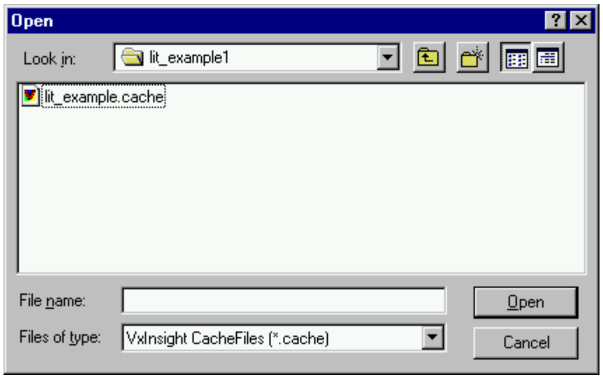

2. To view a sample data set, click on **File** on the main menu, and then on **Open**.
3. A dialog box will open to prompt you to choose a dataset. You will see two folders:
   lit_example1
   pat_example1
   with a literature and patent example, respectively.
4. Double-click on the lit_example1 folder. You will see a lit_example.cache file. Double-click on this filename, and VxInsight will open the corresponding data set.
5. Information on how *VxInsight* displays data and how to navigate data is given in the 'GENERAL OVERVIEW' and 'NAVIGATION AND ANALYSIS' sections of the manual.

GENERAL OVERVIEW

The *VxInsight* Window

The *VxInsight* window consists of the *Main Menu*, the *Side Menu*, the *Main Graphics Window*, the *Object Information View*, and the *Query Menu*.

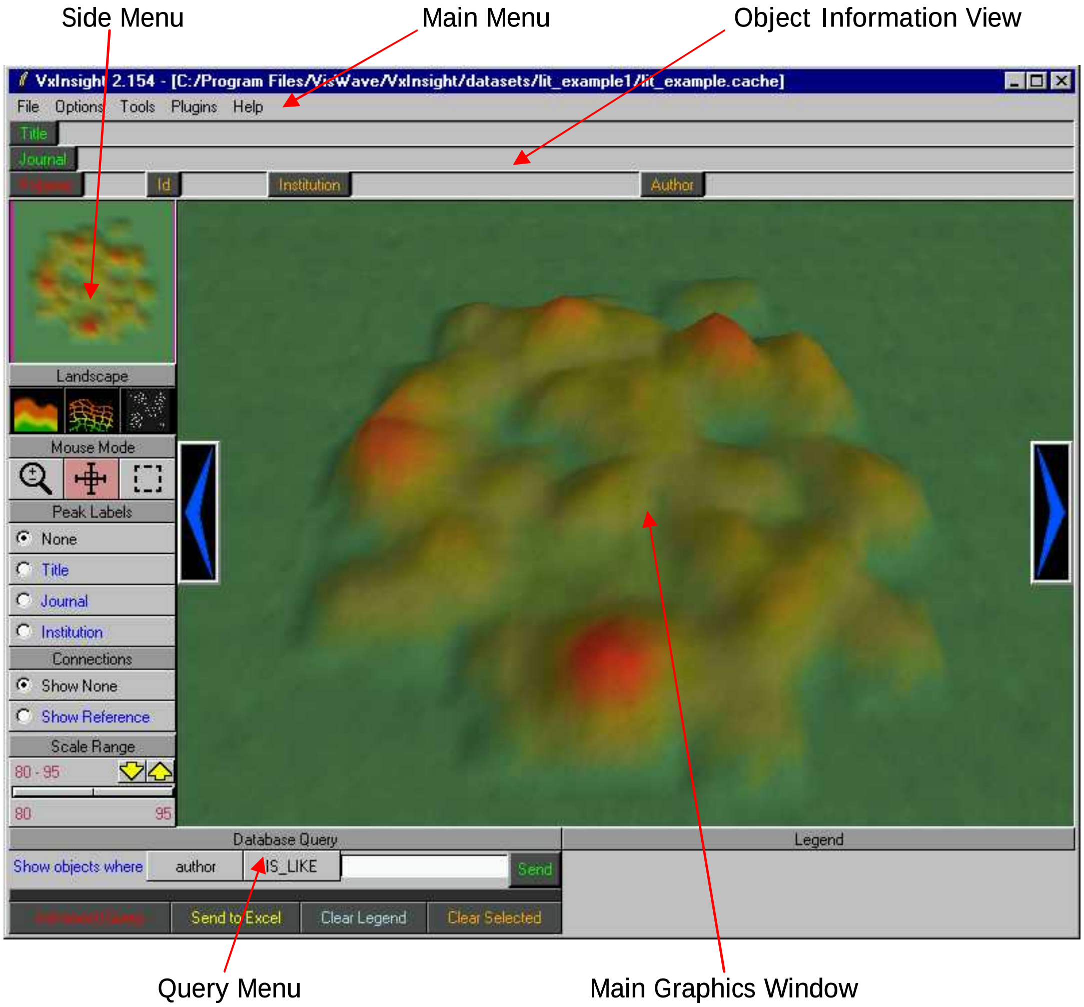

©VisWave, LLC, 1997-2004

Main Menu

Upon startup, the title bar on the application window will contain the current *VxInsight* version number (in the graphic below, 2.154), as well as the path of the currently open dataset. Please use the version number whenever contacting VisWave support.

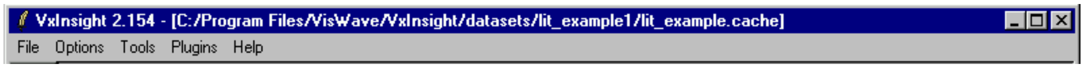

The main menu contains the following menus:

File menu items
- New – creates a new cache file – see 'Creating a Cache File' section
- Open – opens a *VxInsight* dataset by allowing the user to choose an existing <dataset>.cache file
- Save – re-saves the current cache file
- Save As – saves the current cache file to a new user selected <dataset> name
- Save Image As – saves the view in the *Main Graphics Window* to a JPG file
- Exit – exits *VxInsight*

Options menu items (default values are **bolded**)
- Meta Data – **show** or hide the *Object Information View*
- Labels – quick, **medium**, or good precision for labels. Quick labels are less precise, but allow for faster zooming in very large data sets (>10k data objects). Medium labels are more precise, reduce the speed of zooming, and are a good choice for medium sized data sets (2-5k data objects). Good labels are the most precise and are very good for small data sets (hundreds of data objects).
- Graphics – very high, high, medium, or low quality textures in the *Main Graphics Window* – default depends on the graphics speed of the user's computer
- Auto Scale – selects between relative (**on**) or absolute (off) scaling of the peaks in the *Main Graphics Window*

Tools menu items – applications (including some standalone) that can be run from within the *VxInsight* environment.
- Compute Similarity – currently unavailable
- Ordinate – calculates x,y coordinates for data objects using the *VxInsight* ordination routine, *VxOrd*. Requires a *.sim file as input.
- Import Coordinates – imports an x,y coordinate set into the currently open dataset. This feature is very useful when comparing multiple ordinations of the same data.
- Edit Configuration Files – opens an editor where the user can edit the <dataset>.db, <dataset>.config, or <dataset>.stop files. Alternately, these files can all be edited with Windows Notepad, Wordpad, or any other word processing program.

Plugins menu items will vary with the specific plugins installed. When a plugin is selected, a new main menu item corresponding to the plugin will appear.

Help menu items
- Web – opens the user's default browser to the VisWave help page

Side Menu

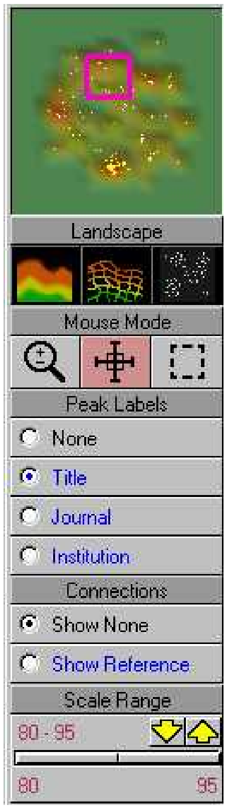

Global View Window – also known as the "you are here" box. This view shows a user what portion (contained within the magenta rectangle) of the entire landscape is currently being displayed in the *Main Graphics Window*. This feature is most beneficial when zooming and rotating the landscape in the main graphics window.

Landscape Modes – three icons that control the display mode in the *Main Graphics Window*. From left to right, the modes are **Terrain View**, **Wireframe**, and **Scatterplot**.

Mouse Modes – three icons that determine the function of mouse clicks in the *Main Graphics Window*. From left to right, the modes are **zoom**, **pick**, and **drag box**. When the zoom mode is active, a mouse click in the *Main Graphics Window* will zoom the landscape in (left-click) or out (right-click). When the pick mode is active, a mouse click on a data object in the *Main Graphics Window* will cause metadata to display in the *Object Information View*. When the drag box mode is active, the user can draw a drag box around a set of data objects in the *Main Graphics Window*.

Peak Labels – a toggle menu used to select the field to use for peak labeling. The menu is generated using field names designated by a user in the <data set>.config file as discussed in the 'Datasets and File Structure' section. Selection of the *'None'* option turns off the peak labels.

Connections - a toggle menu used to select the type of relationship links (such as patent citations) to be shown in the *Main Graphics Window*. Links are shown as arrows between pairs of data objects. Multiple types of connections can be defined. The menu is generated using field names designated by a user in the <data set>.config file as discussed in the 'Datasets and File Structure' section. Selection of the *'Show None'* option turns off all connections.

Range – allows a user to narrow the data set to a specific range of information. The range is narrowed or broadened by clicking on the yellow down or up arrows, respectively. The slider bar can be moved to the right or left with the cursor to move the active range. Upon start-up of a data set display, the range is determined if a range field has been defined in the <data set>.db file as discussed in the 'Datasets and File Structure' section.

©VisWave, LLC, 1997-2004

Main Graphics Window

The *Main Graphics Window* displays data in a Terrain View, Wireframe, or Scatterplot view, as selected in the Landscape modes portion of the *Side Menu*. Pictures of the different views are shown below.

Terrain View Mode

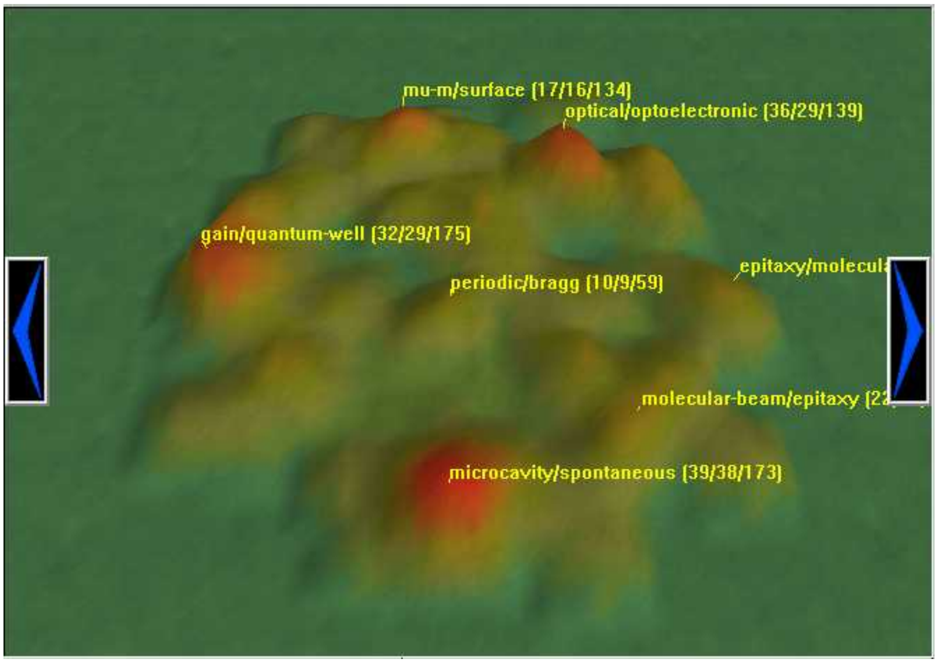

The mountains in the terrain view are based on the density of the data objects on the plane beneath the mountains. The height and shape of a mountain are determined by the number and position of the data objects within a specified radius on the plane. The color of the mountains does not contain any additional information, but serves to accentuate visual perception of the height of a peak.

Peak labels are shown in the *Main Graphics Window* for many of the peaks in the landscape. The labels are based on metadata, and serve to give the user a general overview of the content of different peaks within the context of the entire landscape. Labels show the two most common values found in a chosen data field (chosen in the peak labels are of the *Side Menu*) for all of the data objects within a certain screen radius.

The label format is *<most common value>/<second most common value> (#/#/#)*, where (#/#/#) is defined as (number of occurrences of first value/number of occurrences of second value/total no. of data points sampled). For the bottom-most peak in the figure above, *labeled microcavity/spontaneous (39/38/173)*, this means that the words "microcavity" and "spontaneous" occurred 39 and 38 times, respectively, in the titles of the 173 documents used to generate that label.

Wireframe Mode

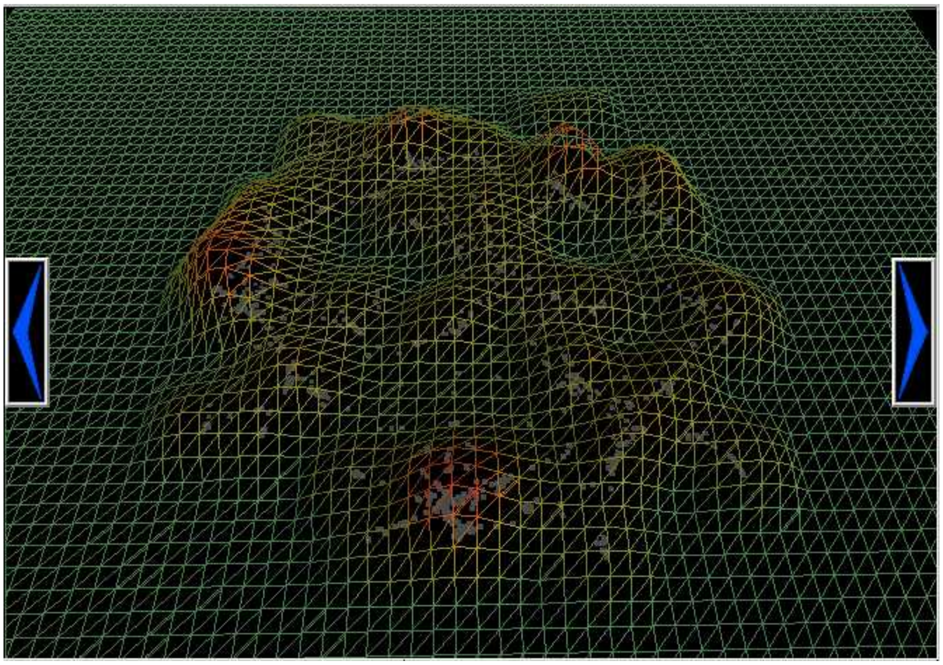

The wireframe mode shows the peaks as a wireframe (compare the peak shapes and sizes to those in the terrain view on the previous page), while showing the individual data objects as gray dots on the plane.

Scatterplot Mode

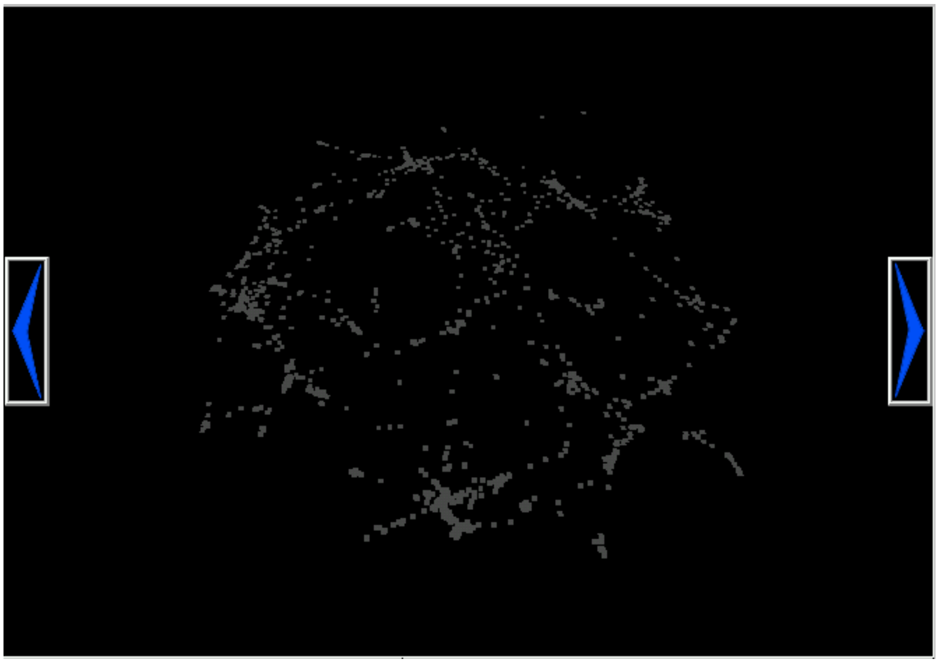

The scatterplot mode shows the individual data objects as gray dots on the plane without showing any mountains. The aspect of the views in the *Main Graphics Window* is not from directly above the data, but is rather from about 45 degrees above the plane. Thus, the scatterplot view is will not be the same as if you were to plot the data in Excel, but is narrowed at the far end.

Zooming and Rotating

Zoom In

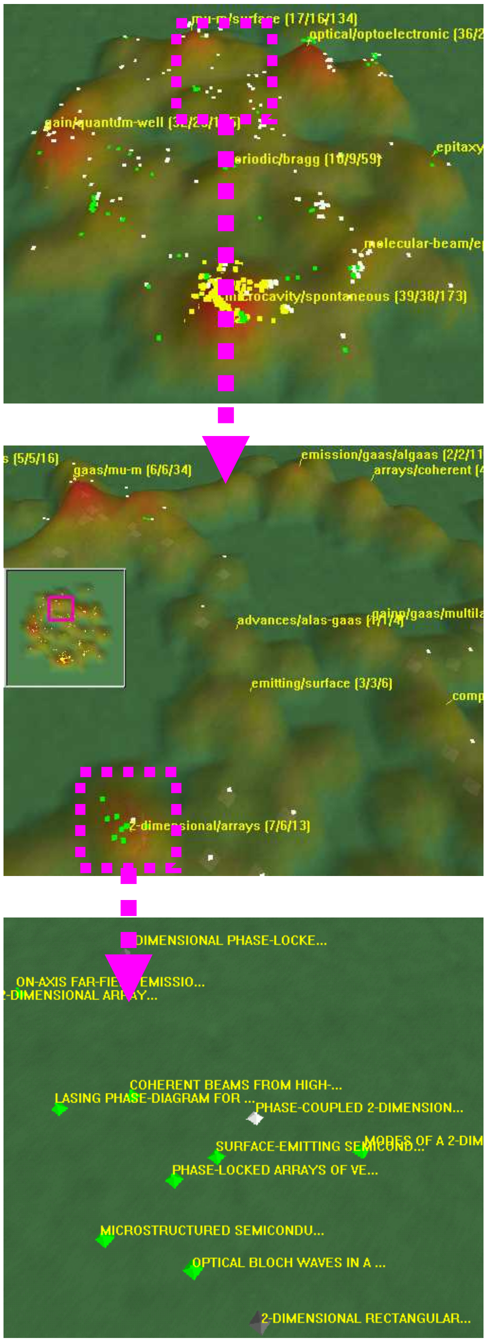

The left-mouse button click allows the user to zoom in to an area of interest. The user may click for a controlled zoom or may choose to hold the mouse button down for a faster zoom. Each zoom step will tend to move the portion of the landscape pointed at by the cursor to the middle of the *Main Graphics Window*. As the user zooms in, the terrain label generation reflects the newly created peaks. Also, once the user has zoomed in to where only a few objects are shown in the *Main Graphics Window*, each object will change from a dot to a larger pyramid. This change is to make it much easier for a single object to be selected (see Select Single Data Object).

Zoom Out

The right-mouse button click allows the user to zoom back out. The user may click for a controlled zoom or may choose to hold the mouse button down for a faster zoom. As the user zooms back out, the dynamic label generation again reflects the newly created peak information.

Rotating

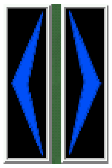

There are two arrows supplied for rotating the landscape to the left (counterclockwise) or to the right (clockwise). These arrows are at the left and right sides of the *Main Graphics Window*. The user simply places the mouse pointer on an arrow and the landscape will begin to rotate in that direction.

Select Single Data Object

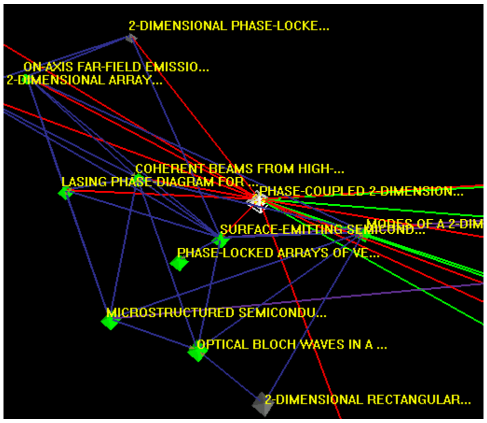

A single data object can be "picked" or selected either by left-clicking on the object (one of the gray dots or gray pyramids) in Pick mode 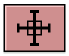 or by SHIFT-left-clicking on the object in Zoom mode 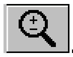. When the objects are represented as pyramids, the selected object will be shown as the one with a larger white pyramid surrounding the object pyramid. In the figure to the right, the object in the middle (labeled PHASE COUPLED 2-DIM...) has been selected.

When an object is selected with connections shown (see Connections in the Section on the Side Menu), the connections into and out of the selected object change colors to red (incoming) or green (outgoing), respectively. This makes it easy to see the connections between the selected object and other objects.

Select Data Objects Subset (Drag Box Selection)

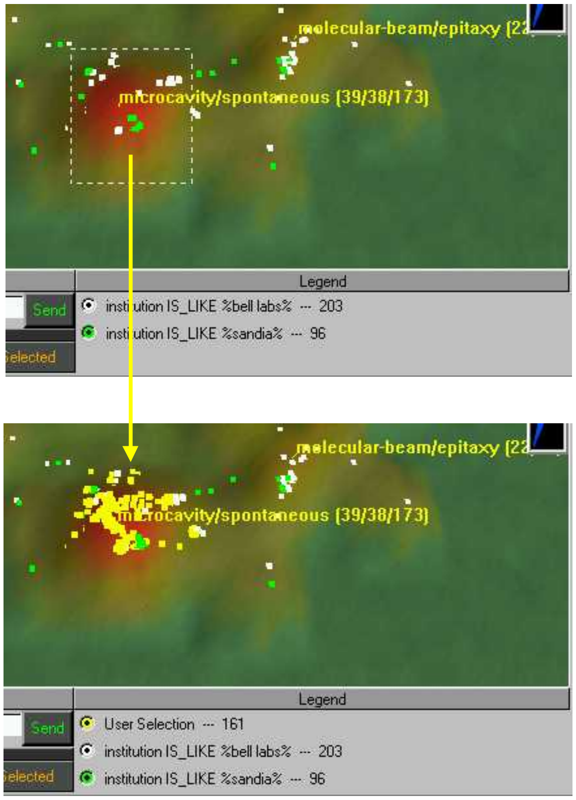

An area of interest within the landscape can be selected and marked in the drag box mouse mode 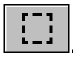. After clicking on the drag box mouse mode button, simply use the cursor to drag a box (shown as dashed white lines) around the area of interest. All of the data objects within the area of interest will be marked with a colored dots, and a new "User Selection" legend entry will appear in the legend. User selection legend entries are treated as any other legend entry.

©VisWave, LLC, 1997-2004

Object Information View

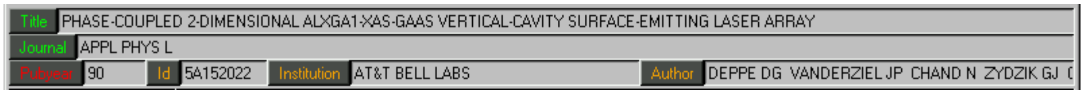

When a data object is selected in the manner described in the Select Single Data Object section, metadata for that object is retrieved from the database and fill the fields in the *Object Information View*. This view can be configured in the <data set>.config file as discussed in the 'Datasets and File Structure' section.

Query Menu

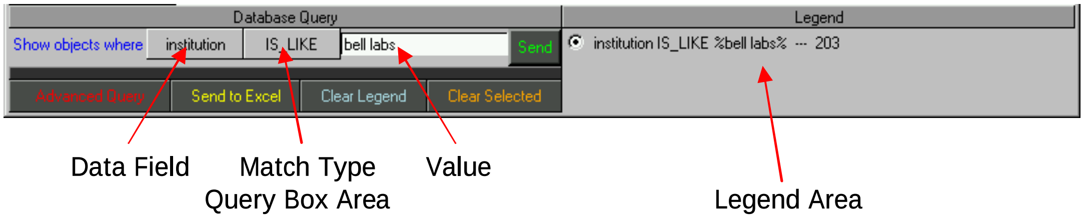

The Query Menu is composed of the query box area and the legend area.

Query Box

The query box area allows the user to query fields in the database for data objects containing specific information. Queries can be made to any field defined in the <data set>.db file as discussed in the 'Datasets and File Structure' section. Data objects that are matched by the query are marked with a colored dot on the landscape. The legend area shows the queries that have been made with their corresponding color markers.

Queries are constructed and performed in four steps:

- First, the user selects the desired Data Field from a pull-down menu that opens when the **Data Field** button is held down. In the figure above, the field "institution" has been selected.
- Second, the user selects the desired Match Type from a pull-down menu that opens when the **Match Type** button is held down. In the figure above, the match type "IS_LIKE" has been selected. The Match Type selection options are:
  IS_LIKE – used for text and assumes wild-cards on both ends of the value string
  IS_EXACTLY – used for text with no wild cards
  >, >=, =, <>, <=, <   are the numeric operators they appear to be, and should be used with numeric fields. If used with text fields, these match types can give interesting results. If used with numbers appearing in text fields, the numbers are treated as ASCII characters (e.g. ordered like 1, 10, 11, 100, 2, 3 rather than 1, 2, 3, 10, 11, 100) and will not give numerically accurate results.
- Third, the user types the query value into the **Value** box. In the figure above, the query value is "bell labs". The entire query string is thus equivalent to the SQL query SELECT ID where institution like '*bell labs*'
- Fourth, the user presses the **SEND** button located to the immediate right of the Value box.

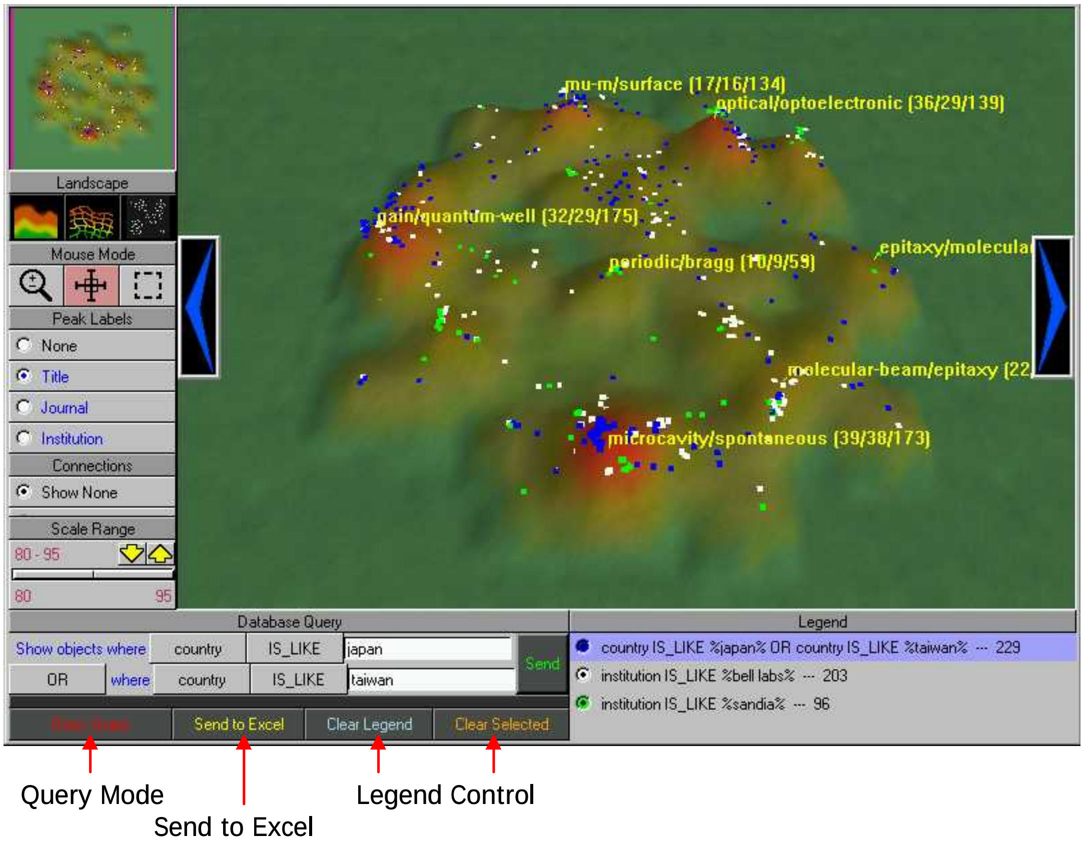

Legend
The legend area shows the queries that have been made with their corresponding color markers. Each item in the legend is represented with a color that associates all the data objects on the landscape meeting the specified criteria. Specific data objects that have met criteria for more than one query have multiple dots showing the multiple queries that were matched.

A maximum of eight (8) queries can be performed and held in the legend. After reaching this limit, a query must be cleared or deleted before another can be performed.

There are three button that control the legend and associated functions:

- **Clear Legend** – Clears all of the legend entries. All color markers on the landscape that are associated with the legend entries will also be cleared.
- **Clear Selected** – Entries on the legend can be selected by clicking on them. When selected, a legend item will be highlighted blue, as shown in the first legend item in the figure above. Multiple legend items can be selected. Clicking on the Clear Selected button will clear the selected legend entries and all of their associated color markers on the landscape.
- **Send to Excel** – Retrieves data from the database for the objects corresponding to the selected legend entry, starts Excel, and places the data in an Excel spreadsheet. Only one legend entry can be selected at a time for this function.

Query Mode
There are two types of database queries offered by VxInsight. One is a single or "basic" query and the other is a multiple or "advanced" query. The advanced query is sometimes

referred to as a "boolean" query.[2] A user can switch between "basic" and "advanced" mode using the **Query Mode** toggle button.

In the advanced query mode, shown in the figure on the previous page, the steps performed by the user are nearly the same as for a basic query. Selection of the Data Field, Match Types, and Values is the same as for the basic query, but is done for each half of the advanced query.

In addition, a boolean operator must be chosen by clicking the AND/OR button. The three supported operators are:

- AND – both values specified for the fields selected must be contained in a data object in order for it to be included in the resulting information
- OR – either value specified for the fields selected must be contained in a data object in order for it to be included in the resulting information
- AND NOT – the first but not the second value specified for the fields selected must be contained in a data object in order for it to be included in the resulting information

[2] Only a two-field query is supported at this time.

AUTOMATED DATA IMPORT

VisWave provides a data import module, VxImport, which will import new data sources automatically with just a few mouse clicks. Every licensed user of VxInsight will receive the VxImport software along with customization for their specific data sources. Please contact VisWave for more details on customization.

VxImport

VxImport is a program designed to read a users input files (tab delimited, XML, or general tagged record files), and automatically imports those files into VxInsight. VxImport creates the Microsoft Access database, DB, CONFIG, STOP, and SIM files, generate coordinates, and creates the VxInsight CACHE file for a dataset.

The installation of VxImport follows the same pattern as the installation for VxInsight that is explained in the Getting Started section of the users manual, and will place a VxImport icon on your desktop.

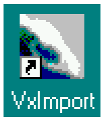

To start VxImport, double-click on the VxImport icon on your desktop. The VxImport program screen will appear. The steps to using VxImport are as follows:

Step 1: Select Import Type

Select the import type for your type of data. You can select tab-delimited, XML, or one of the tagged record formats (note: if you need a specific format supported please contact VisWave support). The style sheet will be set automatically. (In the future users will be able to save their own custom style sheets but this function does not currently exist.)

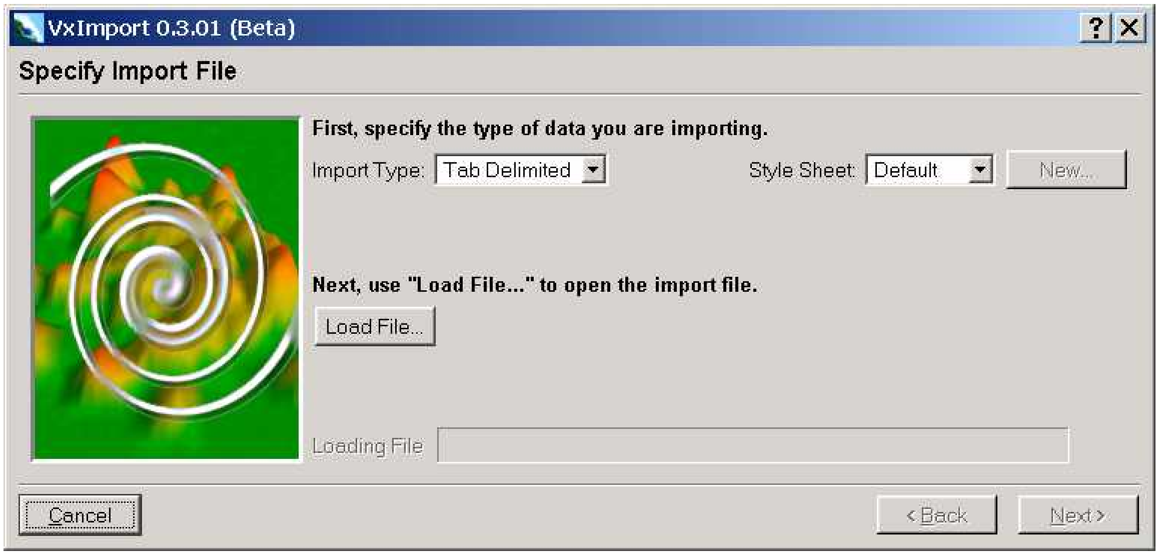

Step 2: Load your data file.

You can select the file you want to import by hitting the 'Load File...' button. A dialog box will appear and you can select the file you want to load. While loading the file the progress bar will indicate the progress of the load. When the loading is finished the 'Next>' button will become active. To proceed hit 'Next>'.

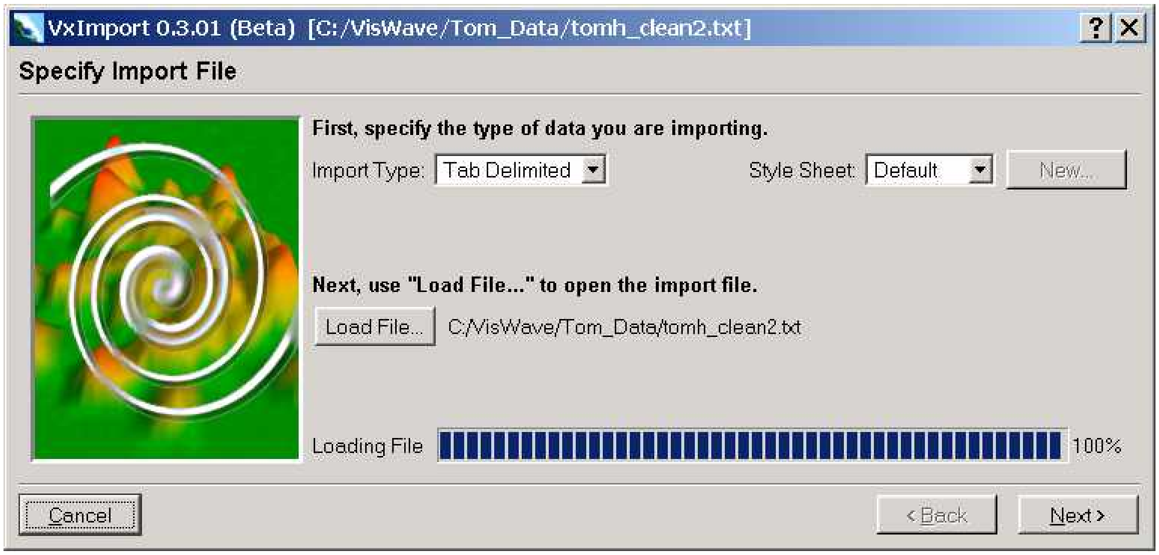

Step 3: Data properties

The next window in the import wizard allows the user to set up the properties of the data they are loading. This window has a lot of options so we will go over them one by one.

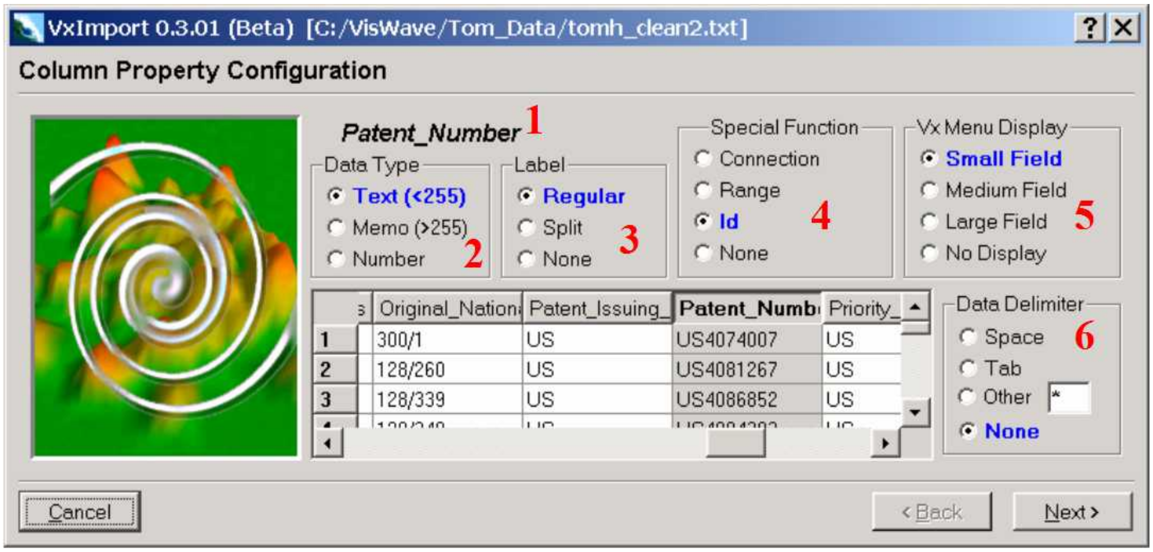

1. **Column Label:** Shows the currently selected column. All changes made to the data properties will affect this column only.

2. **Data Type:** Select the proper data type for the column. If the column holds data that is less than 255 characters select **Text** (for instance, author names or country). If the column holds data more than 255 characters select **Memo** (for

instance, abstract or claims). If the column contains an number (like pub year) than select **Number.** In general you can usually leave most columns as **Text** or **Memo** (dates are **Text** 12/3/2003).

3. **Label:** This options controls how labels above the mountains are shown. **Regular** will keep all the words in the column together when making the label and **Split** will 'split' up the words before making the label. **None** will mean that you do not want to see labels for this column.

VxInsight creates peak labels by running through the data objects underneath the peak and pulling out the most common words or phrases from the data. If you select Regular than the data for that column is treated as a phrase and not broken up, if you select Split than the column data will be broken into words and the most common words will show up as the peak label.

An example will illustrate the use of the label options. When looking at a patent dataset lets say you have three different columns of data, a title, an assignee, and an id. If you select the **Regular** option for title than the words will not be split up before creating the mountain label. You will get peak labels that are very long and not very informative. If you select **Split** the labels will contain the most common words and will hopefully be much more informative about the data each mountain contains.

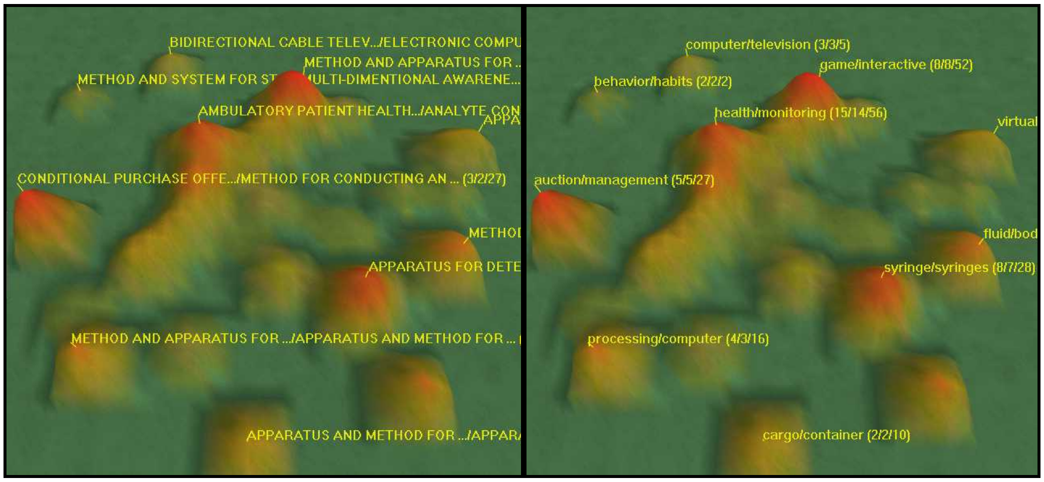

Using Regular and Split as label options on the title field.

Okay continuing with our example. Now your deciding what type of label to use for the assignee column and you first choose **Split**. In this case the Assignee data will get split into words and the most common will be used for labels. So you will get very odd labels like 'International' when it's more informative to get a label of 'International Business Machines'. For column data like Assignee it's better to keep the words together. The last option is **None** and this is used because there are often many fields where you don't care to see a label or it doesn't make sense.

©VisWave, LLC, 1997-2004

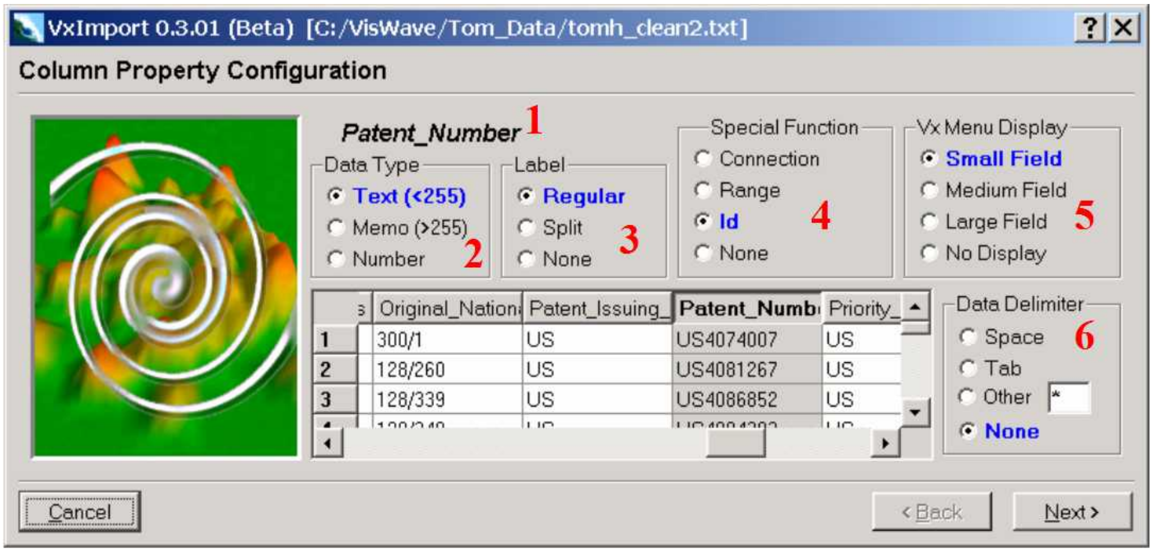

4. **Special Function:** This option allows the user to indicate that this column has one of the special functions. The last function **Id** is straightforward; it indicates that this column contains the unique id for this record. The **Connection** function is used when a column contains data with the id's of other records (for instance references or citations). Using the **Connection** function means the user will be able to see 'links' between the data objects in VxInsight. The Range function will allow users to pick a certain range in VxInsight and only see data within that range (for instance publication year or issue year). **Note:** Any column selected for range data must contain only integers (e.g. 12/3/2003 or 123.5 are NOT acceptable data for ranges)

5. **Vx Menu Display:** VxInsight can display your data in several different ways.

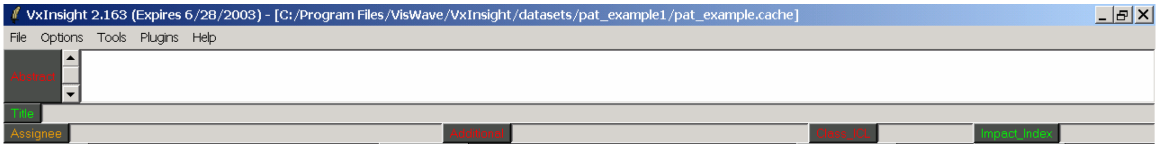

The picture above shows the three options. The first row is a **Large Field** display for things like abstracts and claims. The second row is a **Medium Field** display for data that usually fits on one line, like titles. The third row is four **Small Field** displays for data that needs less than one line, like issue date, assignee, etc.

6. **Data Delimiter:** If the column contains several pieces of data, like references separated by tabs, then specify what the data delimiter is. Normally just leave this option as **None**.

Step 4: Specify Mapping Field

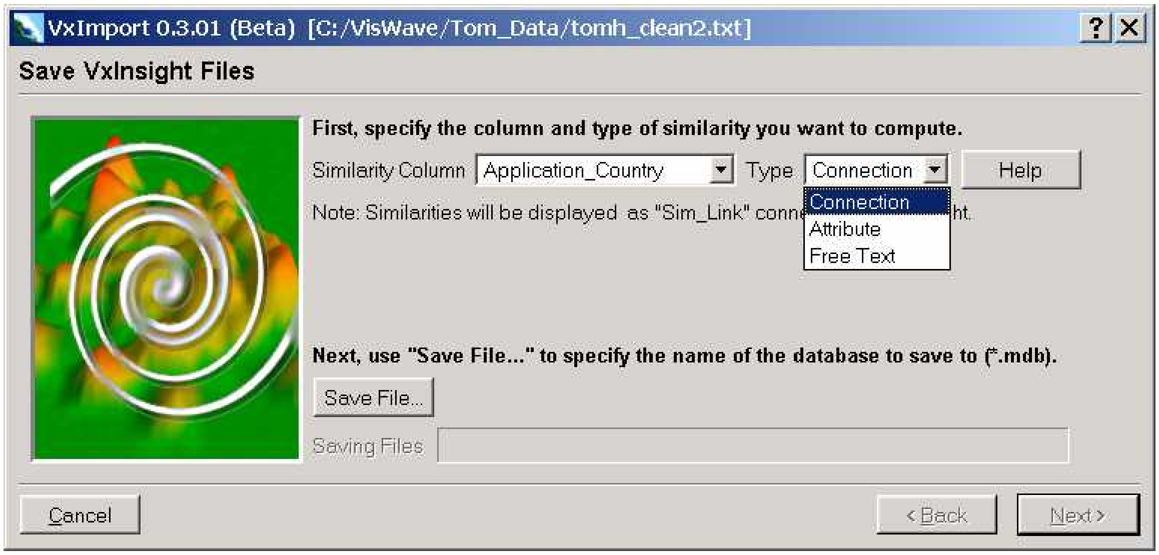

See the help button for help on which type of similarity you should choose based on your type of data.

Step 5: Save the dataset file

After specifying the column of data and the type of similarity you want to compute, you now specify what filename you want to save your data under. NOTE: A current limitation of VxImport is that you cannot save your file to a path that contains a "space". For instance **c:\program files\datafile\foo.mdb** will NOT work. (We are currently working on this bug).

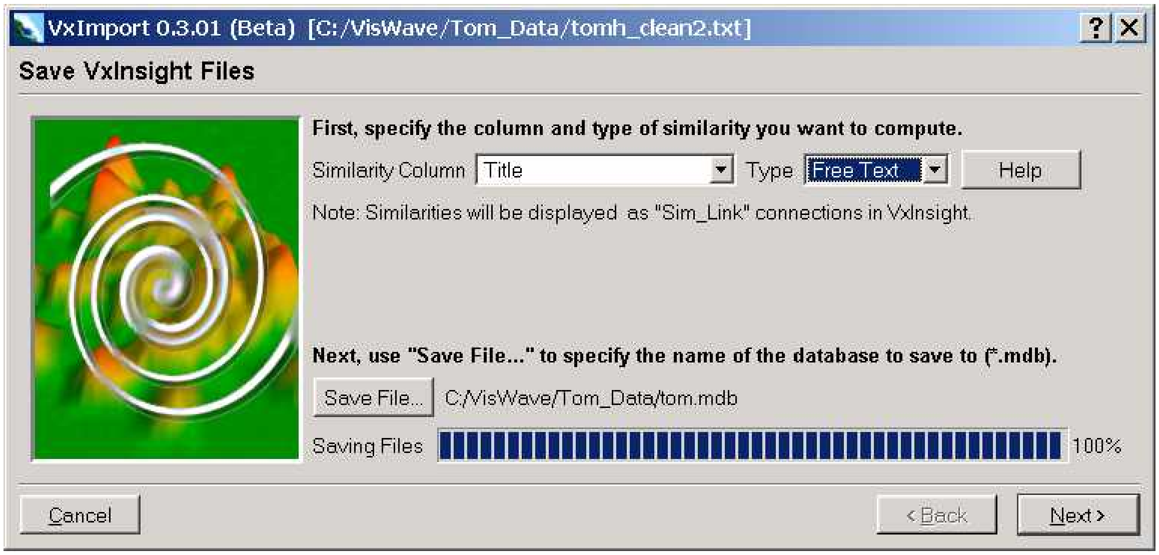

Step 6: Communicate with VxInsight

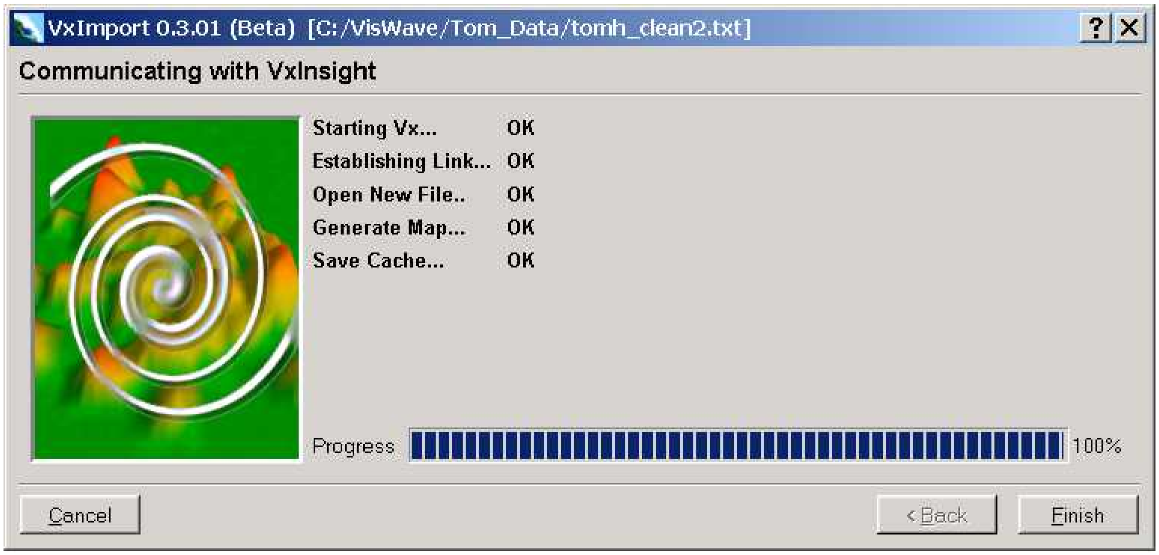

After you save the dataset files, the next panel will communicate with VxInsight and bring up your dataset. This process can sometimes take a while based on the size of the data you are importing. VxInsight will appear do some computations and then a map of your imported data should come up.

MANUAL DATA IMPORT

For users that want a more "hands-on" experience with their data, or who desire to use more advanced similarity measures than are available in VxImport, we describe the manual data importation process here in detail. This section should also allow the user to understand the steps that are being performed by VxImport, and give information that will allow the various VxInsight files to be easily modified if necessary.

Getting your data ready to view and explore in VxInsight requires that your data be available in a location that is accessible via an ODBC driver. Common examples of this are a Microsoft Access database or a Microsoft Excel spreadsheet. In addition to data accessibility, there are several processing steps that must take place to make it so your data can be viewed in the landscape format used by VxInsight. In general terms, these steps are as follows:

- Create the additional files needed by VxInsight to access your data.
- Specify a similarity between data objects (sometimes known as a similarity function or a distance measure) that can be used to group objects.
- Generate [x,y] positions for each data object from the similarity metric; this step is known as ordination.
- Load the data into VxInsight and create a CACHE file.

These steps can be done automatically with the VxImport program, or they can be done manually as described below.

Required Files

For most situations, a dataset configured for use with *VxInsight* will require five files for operation:

1. A file containing the data, typically a Microsoft Access file or Microsoft Excel file.
2. A database alias file with extension <dataset>.db.
3. A VxInsight configuration file with extension <dataset>.config.
4. A stopword file with extension <dataset>.stop.
5. A local cache file with extension <dataset>.cache.

The last four of these files listed above must have the same <datset> name (e.g. lit_example.db, lit_example.config …) for VxInsight to run properly. VxInsight can run without the stopword file, generating a nonfatal error message.

If you are connected to a network (or the Internet) while running VxInsight, and you want your data to be hyperlinked to original or ancillary sources of data, this can be accomplished with a <dataset>_click.tcl file. An example of this file type is given in the pat_example1 sample data set. The file pat_example_click.tcl contains information that will start your default browser and bring up the US Patent and Trademark Office full text web page for any patent in the dataset given that the US patent number (e.g. 6012345) is used in the ID field.

©VisWave, LLC, 1997-2004

Your Data Source

Microsoft Access

The most typical data construction used with VxInsight is a Microsoft Access file containing several tables and/or queries. All tables containing fields that the user might wish to query from within VxInsight must have a common key (e.g. the **pat_no** field is common to all tables in the figure below).

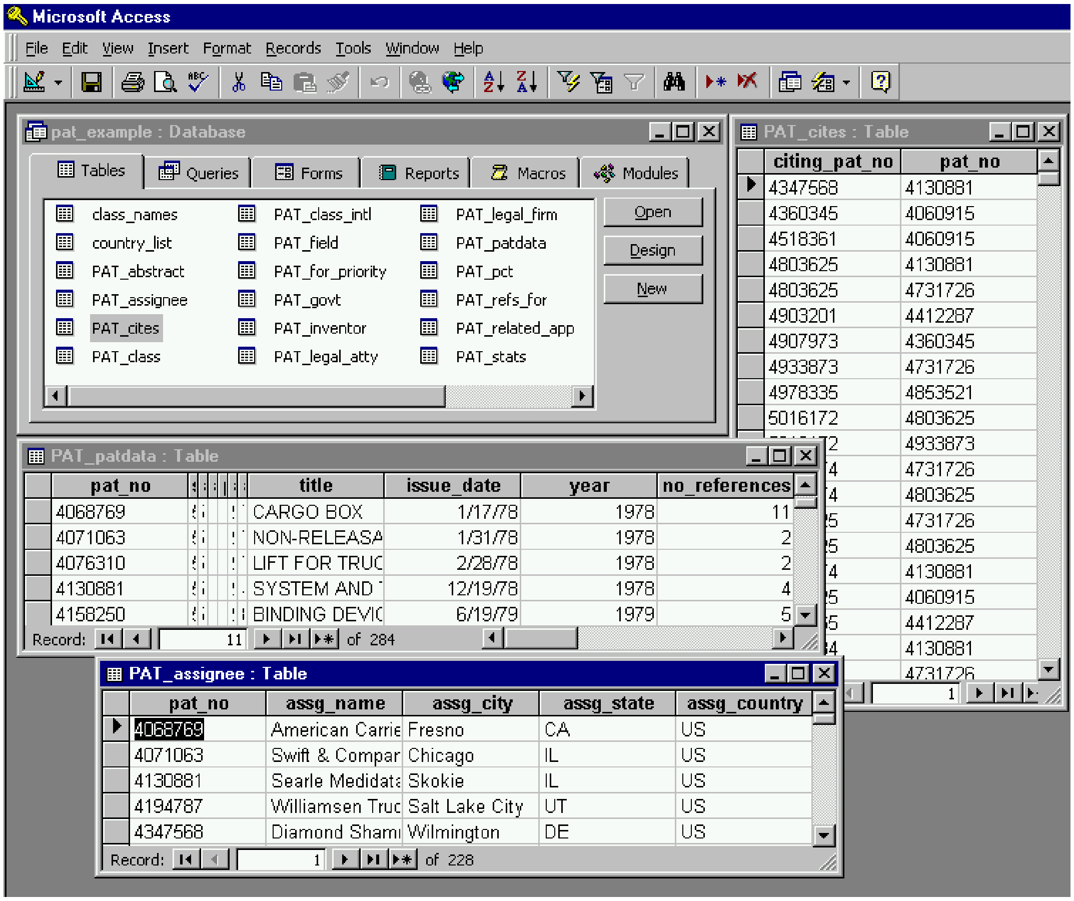

Microsoft Excel

You will be able to see your Microsoft Excel data in VxInsight, but some of the queries will not work. The recommended approach is to move your data from Excel into Access or some other fully ODBC compliant database. (See the section "Converting Excel data to Microsoft Access")

In order to view data from a Microsoft Excel file in VxInsight the following preparation to the Excel file must be made.

- The Excel file must have a column that is a unique identifier (similar to a database key). Make sure that the field used as the unique identifier was brought into Excel as a 'text' field, not as a 'number' or 'general' field.
- Each column must have a 'Header' in the first row that describes the column.
- Select all fields in the excel file and give it a 'name'. This is done by selecting all fields with data, then performing the menu operation *Insert->Name->define…*

- Within the *define* function, click on "Add" and call it 'mytable'
- Save Excel file and quit Excel

Converting Excel data to Microsoft Access

This sequence is by no means a replacement for standard literature on the operation of Excel or Access, but is intended to give a few helpful tips for the novice user.

There are 10 easy steps for moving data from Excel into an Access database.

1. Open Access.
2. Create a new 'blank' database. Remember the name you give the database. You will need it later.
3. Menu: File -> Get External Data -> Import…
4. Change the file type to 'Excel (*.xls)' at the bottom of the browse window.
5. Find your excel file.
6. You will then have a series of dialog boxes that ask about your data and which fields you want to bring into the database. Be sure to read the options carefully.
7. Store your data 'In a New Table'.
8. Next it will ask you to put field names on the data you are importing. (Remember these field names. You will need them later). You should designate most fields as 'text' fields in Access, including the field containing unique identifiers for your data objects. Only numerical fields that will be used in numerical queries (e.g. <, <=, =, etc.) should be designated as numerical fields.
9. Next you will be asked about primary keys. VxInsight does not require that you let Access add the primary key.
10. On the final dialog box you can change to name of the table to which the data will go. We suggest you change this name to 'mytable' so that it will be the same as our documentation examples.

Converting Text data to Microsoft Access

This help file is by no means a replacement for standard literature on the operation of Access, but is intended to give a few helpful tips for the novice user.

There are 12 easy steps into getting text data into an access database.

1. Make sure all data in your text file(s) is tab delimited.
2. Open Access.
3. Create a new 'blank' database. Remember the name you give the database. You will need it later.
4. Menu: File -> Get External Data -> Import…
5. Change the file type to 'Text Files (*.txt)' at the bottom of the browse window
6. Find your text file.
7. You will then have a series of dialog boxes that ask about your data and which fields you want to bring into the database. Be sure to read the options carefully.
8. When you are asked about field names you should pick descriptive names and write them down for later. If you are creating multiple tables in access make sure they share a unique identifier field that is the same for all tables (e.g. all tables have a field called 'id' that is the unique identifier for the data).
9. Store your data 'In a New Table'.
10. Next it will ask you to put field names on the data you are importing. (Remember these field names. You will need them later). You should designate most fields as

©VisWave, LLC, 1997-2004

‘text’ fields in Access, including the field containing unique identifiers for your data objects. Only numerical fields that will be used in numerical queries (e.g. <, <=, =, etc.) should be designated as numerical fields.

11. Next you will be asked about primary keys. VxInsight does not require that you let Access add the primary key.
12. On the final dialog box you can change to name of the table to which the data will go. We suggest you change this name to ‘mytable’ so that it will be the same as our documentation examples.

The VxInsight DB file (<dataset>.db)

Once your data is in an ODBC-compliant database, you will need to create several other files that will allow VxInsight to display your data.

The VxInsight DB file (named <dataset>.db) is the file that tells VxInsight where to connect to your data. Steps to create the VxInsight DB file are as follows:

1. First, open your database file and write down all the tables and fields that you will want to have access to from within VxInsight. Any mistakes in either the table or field names will make it so that data cannot be accessed from within VxInsight.
2. Create a new file in notepad or another text editor and call it <dataset>.db
3. Create the entries as shown below. Each field that you wish to be able to view or query from within VxInsight should be listed on a separate line in the DB file. Save the file.

The VxInsight DB file is a tab-delimited file that contains the following three columns:

- **First column**: Data nickname (note that the first nickname **must be called 'id'** and must point to the database field containing the unique identifiers for your data objects. All other nicknames are arbitrary, but should be named intuitively.
- **Second column**: Location of data field with format

  ```
  autoconnect*<datafilename>::table_name::field_name
  ```

  Optionally, if you are not using a Microsoft Access database you can specify 'DIALOG' for the data filename. The software will bring up a dialog box allowing you to chose the data source.
- **Third column:** designation of the field as either CACHE, CONNECTION, or NO. The **id** field must be have a cache designation. All other fields that you wish to use for peak labeling should also have a cache designation. The connection designation is used to show references, such patent or literature citations. Multiple cache and connection designations can be used. All fields not to be used as peak labels or to show connections should have a NO designation.

One special field that is used in the DB file is the **Range** field. The range field designates which data field is to be used on the Range Slider that can be seen on the Side Menu of the VxInsight window. The most typical field used with Range is the Year field, especially for patent and literature data.

Below is an example DB file.

```
Id              autoconnect*lit_example.mdb::article::icf              cache
Range           autoconnect*lit_example.mdb::article::pubyear          cache
Title           autoconnect*lit_example.mdb::article::title            cache
Journal         autoconnect*lit_example.mdb::article::journal          cache
Institution     autoconnect*lit_example.mdb::address::org              cache
Pubyear         autoconnect*lit_example.mdb::article::pubyear          no
Country         autoconnect*lit_example.mdb::address::country          no
Author          autoconnect*lit_example.mdb::author::auth_name         no
Reference       autoconnect*lit_example.mdb::cites::citing             connection
```

©VisWave, LLC, 1997-2004

The VxInsight config file (<dataset>.config)

The VxInsight config file (named <dataset>.config) is the file that tells VxInsight what fields to place in the *Object Information View* and which features to use as labels in the *Side Menu*. Steps to create the VxInsight config file are as follows:

1. Create a new file in notepad or another text editor and call it <dataset>.config.
2. Create the entries as shown below. You will need to know the nicknames that you used in the DB file. Save the file.

The VxInsight config file is a tab-delimited or space-delimited file that contains the following sections and fields:

First section: This specifies which fields are to be shown in the *Object Information View*. The options are:

```
Add_Large_Info_Field <nickname> lines fieldsize color
Add_Medium_Info_Field <nickname> lines fieldsize color
Add_Small_Info_Field <nickname> lines fieldsize color
Add_Text_Info_Field <nickname> lines fieldsize color
```

These options display data in a small, medium, or large text box, or a floating text window, respectively. The large field can use multiple lines, the medium field is used for single full lines, and the small field places four fields on one line. The additional information (<nickname> lines fieldsize color) designate the field to display using the DB file nickname, the number of lines, the fieldsize (should add to 80 for each line), and label color for each entry.

Second section: This specifies which fields are to be used for peak labels in the *Side Menu*. The options are:

```
Add_Label_Field <nickname>
Add_Label_Field_Split <nickname>
```

The 'split' option will split your field into individual words before labeling, while the first option will use the entire field. Thus, it is most useful to use the split option for fields such as titles and terms, but use the full field for institutions, etc.

Third section: This specifies which fields are to be used for connection fields in the *Side Menu*:

```
Add_Connection_Field <nickname>
```

This option allows you to specify a field that contains a second list of 'id's, such as references or citations. This is useful for showing lines between objects that are 'connected'.

Below is an example file

```
Add_Medium_Info_Field Title 1 80 green
Add_Medium_Info_Field Journal 1 80 green
Add_Small_Info_Field Pubyear 1 2 red
Add_Small_Info_Field Id 1 5 orange
Add_Small_Info_Field Institution 1 30 orange
Add_Small_Info_Field Author 1 40 orange
Add_Label_Field_Split Title
Add_Label_Field Journal
Add_Label_Field Institution
Add_Connection_Field Reference
```

The VxInsight stopword file (<dataset>.stop)

In VxInsight you can specify what fields you want to mountains to be labeled with. Often these fields will contain words that are not informative when shown as labels (e.g. ‘a’, ‘the’, ‘or’, etc…). You can stop these words from appearing as labels by making a stopword file. This file is simply a text file of the same name as the cache file. The text file contains a ‘stopword’ per line. An example is given below.

Listing for <dataset>.stop:

```
a
the
an
or
such
…
```

If you modify your stopfile while running VxInsight (it is often useful to use VxInsight to find technical words that are to common to be good for labeling), you will need to reload your cache file. This is done by choosing FILE > OPEN > <dataset>.cache from the menu.

Construction of a VxInsight sim file (*.sim)

The SIM file is a file containing similarities between pairs of objects and is needed in order to produce an ordination (layout of objects in terms of [x,y] coordinates) using the VxInsight ordination algorithm, VxOrd. Similar objects will be mapped close together and dissimilar objects will be mapped farther apart.

The construction of this sim file may be difficult for some types of data. Some common types of similarities are

- co-occurrence using either categorical or attribute data
- various normalizations based on co-occurrence
- linkage using direct citations, co-citations, or bibliographic couplings
- Pearson's correlation coefficients

VisWave has similarity generators for patent, literature and micro-array data. See the Support and Contacts section for contact information.

The file is a tab-delimited file with 3 columns of the following form:

<**id1**> <tab> <**id2**> <tab> <**similarity**>

The similarity values can be in any range; the VxOrd algorithm remaps the similarities to values between zero and one based on the maximum value in the similarity file.

VxOrd™: Mapping your data

The VxInsight ordination algorithm, VxOrd, is a force-directed placement graph layout algorithm. In general terms, it places similar objects (as defined by the relative values in the SIM file) close together and dissimilar objects far apart.

There are two ways for the user to run VxOrd: from within VxInsight, and in stand-alone mode. When VxOrd is run through VxInsight (using TOOLS > ORDINATE from the menu), the user will be prompted for a SIM file name, and the resulting ordination will be displayed in VxInsight automatically. Note that after running the ordination through VxInsight, the user should SAVE the resulting cache file; otherwise the coordinates will not be saved. When running the ordination through VxInsight, the user is NOT given the opportunity to save an output coordinate file.

The following documentation is for those users who are running VxOrd in stand-alone mode. To run VxOrd, double-click on the VxOrd icon on your desktop.

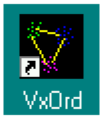

Input files

After VxOrd has been started, the user must open an input file. This is done by choosing either [FILE > Open Sim File] OR [FILE > Open Ref File] from the menu.

VxOrd supports two types of input files: the *sim file (see previous section) and the *.ref file. The *.ref or REF file is a two-column tab-delimited file with format:

<**id1**> <tab> <**id2**>

Given a REF file as input, VxOrd will generate a SIM file using both direct and indirect links between the objects. The order of the two ID columns in the REF file is important in VxOrd. The order of the columns and the resulting similarity type are:

<**cited_id**> <tab> <**citing_id**>	Direct link + Co-citation
<**citing_id**> <tab> <**cited_id**>	Direct link + Bibliographic coupling

Output files

After VxOrd has completed its calculation of the graph layout, the screen turns white and the graph is centered on the screen. At this point you can save two different files, the coordinate file (*.coord) or the statistics file (*.stat).

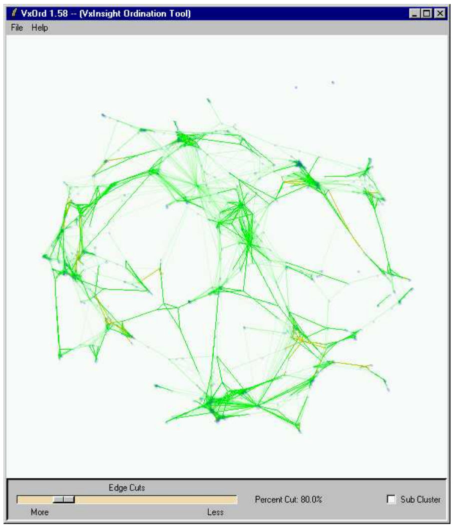

To save a coordinate file (coordinates are needed by VxInsight), choose FILE > Save Coord File from the menu, and enter the name and location of the file that you wish to save. The coordinate file has a format:

<id> <tab> **<x>** <tab> **<y>**

This coordinate file is an ASCII text file and can be opened and viewed (plotted) in other programs such as Microsoft Excel.

The statistics file can be saved by choosing FILE > Save Stat File from the menu. This is a very simple file with information on the number of nodes, edges, and fraction of edges cut during the ordination.

User interaction with VxOrd

The user may want to see the ordination in progress or investigate certain areas after it has completed. The following keys provide navigation within VxOrd.

Left Mouse Button:	Zoom In
Right Mouse Button:	Zoom Out
Arrows:	Move map up, down, left, or right
'c' key:	Center the map on the screen

Advanced features

VxOrd has been optimized for most uses; however there may be occasions where the user will have a preference for more or fewer clusters, or for a more uniform spatial distribution within clusters. The advanced features of VxOrd allow for these types of changes.

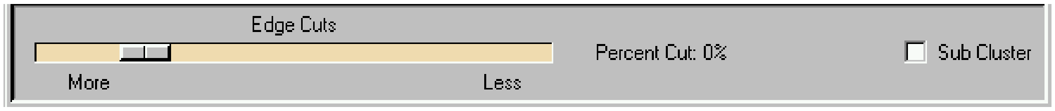

Two types of adjustments are possible in the menu at the bottom of the VxOrd window. Moving the "Edge Cuts" slider will result in more or fewer clusters by telling the algorithm to cut either more or less edges during the ordination.
Selecting "Sub Cluster" will change the final step of the ordination and essentially give a more uniform spatial distribution within each cluster.

As was stated before, VxOrd is optimized for most uses, so any use of the advanced features must be done with great care. In addition, if you use the advanced features, you will want to record exactly what changes you have made (e.g. number of clicks to the left or right on the slider) so that you can reproduce your results if necessary.

©VisWave, LLC, 1997-2004

Creating a Cache File

Once the data are in an ODBC-compliant database, and the DB, CONFIG, STOP, and COORD files have been generated for the dataset, the last step necessary to view the data within VxInsight is to create a CACHE file. The purpose of the cache file is to keep the most used information from the dataset within memory to make the navigation of the data very fast.

Now we outline the final step for pulling your data into VxInsight and creating the reusable CACHE file:

1. Make sure that the <dataset>.db, .<dataset>.config, and <dataset>.stop files are in the same directory
2. Start VxInsight
3. Choose FILE > NEW from the menu
4. Open your <dataset>.db file
5. If all of your settings in the <dataset>.db are correct, you will see a series of informational messages, and then VxInsight will display your data as a single mountain in the middle of the display (all objects are at 0,0 coordinates)
6. To import coordinates into VxInsight, you either
   a) if you have generated a coordinate file, choose TOOLS > IMPORT COORDINATES from the menu, OR
   b) if you have a SIM file, but have not generated a coordinate file, choose TOOLS > ORDINATE from the menu
7. You should now see your data spread out on the landscape
8. Choose FILE > SAVE from the menu to re-save your cache file with coordinates
9. Now that your CACHE file is saved, you can reopen this cache any time you want to view this data.

VxInsight will create a <dataset>.cache file, where the CACHE file has the same name as the DB file. If an existing CACHE file with that name is already present, VxInsight will ask the user if he/she wants to overwrite the existing file before proceeding. If the user chooses not to overwrite the file, the CACHE file process is halted and the user is returned to the previous state.

Note that the TOOLS > IMPORT COORDINATES function is also very useful when you want to compare two different ordinations or maps of the same data. For instance, you can create and save a cache file with one set of coordinates, make several queries or mark (using select data subset) several areas of the map, and then import different coordinates. You can then see where the same queries lie on the new map.

©VisWave, LLC, 1997-2004

Database not found

If the connection to the database specified in the DB file is made without any problem, VxInsight will work as specified in the steps above.

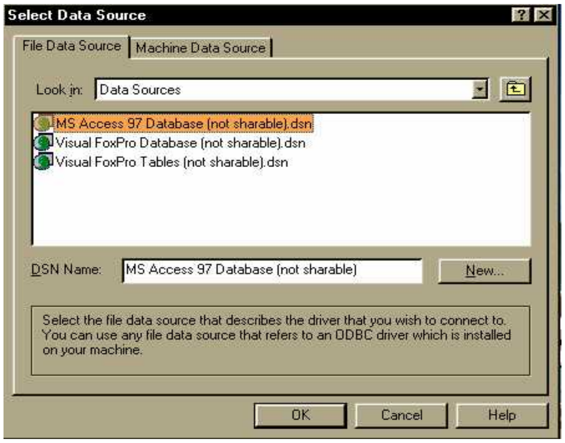

However, if a connection to the database cannot be made (for example, if the user has renamed the Access database file, and has not updated the filename in the DB file), the user will be prompted to find the file or select the data source through Windows dialog boxes. The form of these dialogs depends upon the operating system of the user. One example of the dialog boxes that might appear is shown to the right. Once the data source is selected, VxInsight will continue with its operations and create a CACHE file for the dataset.

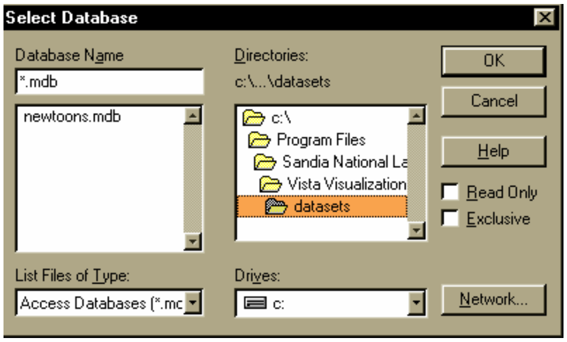

Note that in this case where the database connection is specified manually, VxInsight will not update the DB file with this new information. The user should update the DB file with the appropriate information for subsequent use of the dataset.

With a connection established, VxInsight continues the process and displays informational messages as follows.

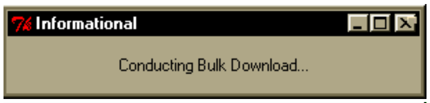

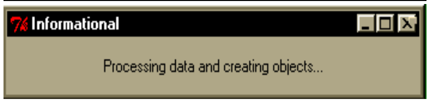

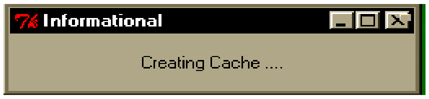

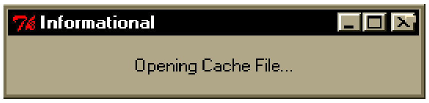

This completes our description of the process required to bring data into VxInsight manually. A front-end reader for VxInsight is currently under development (alpha version) that will automate these steps and create and show maps of your data automatically (see the following section on VxImport). Of course, for more advanced applications, the user may wish to use more sophisticated similarity measures or different layout systems. These operations will still need to be done manually.

SAMPLE ANALYSIS

Coming soon.

PLUGINS

VxGS (VxInsight and GeneSpring)

VxGS is a plugin for the GeneSpring[3] software developed by Silicon Genetics. The installation procedure for VxGS is described in the installation section of this document. As noted in a footnote in the installation procedure, you will not have VxInsight icons on your desktop. The VxInsight functions are made available through GeneSpring.

After selecting a gene list in GeneSpring, you can create a new VxInsight map of the selected gene list. Simply click on the 'Create New Map' icon. VxInsight will start up, process the data, and display a map.

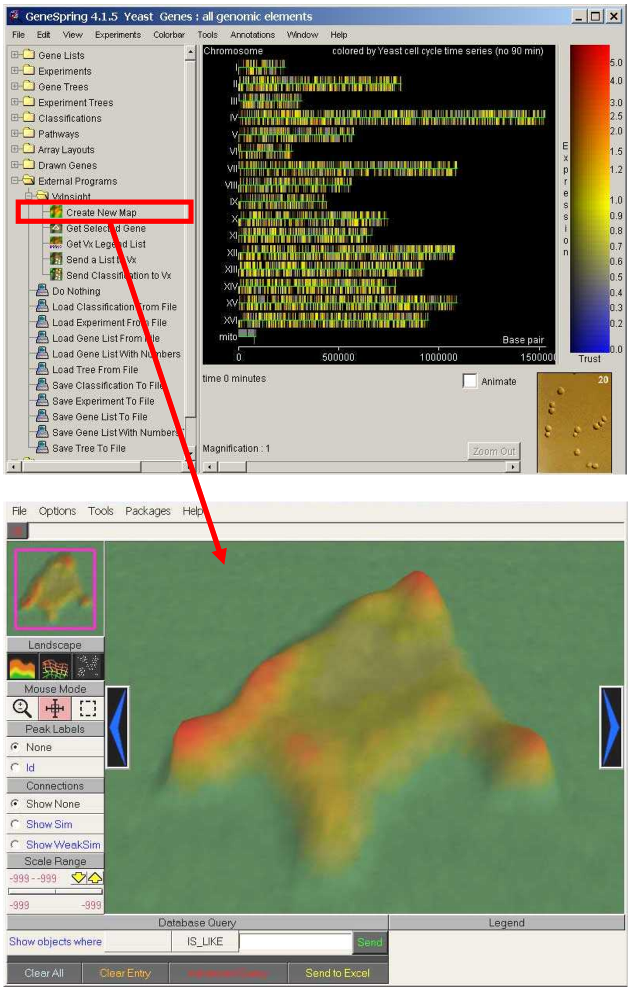

---

[3] GeneSpring is a tradmark of Silicon Genetics.

Passing data between VxInsight and GeneSpring

After creating a new map, a user can now freely transfer data back and forth between VxInsight and GeneSpring. To do this, in VxInsight first select the drag box mouse mode. The user can now select different clusters within VxInsight (see the section on Select Data Objects Subset). Selections show up in the legend (bottom right of image). By selecting one of the legend entries and clicking on the 'Get Vx Legend List' icon in GeneSpring, you now have a new 'Gene List' within GeneSpring. This sequence of actions is represented by the red arrows and boxes in the figure to the right. The new gene list can now be displayed and manipulated like any other gene list within GeneSpring using any of its functions.

You can also send lists and classifications from GeneSpring back to VxInsight where they will be displayed in the legend and color coded on the map. This is shown by the blue boxes and arrows in the figure to the right.

The ability to send any subset (list) of genes back and forth between GeneSpring and VxInsight is very flexible and powerful.

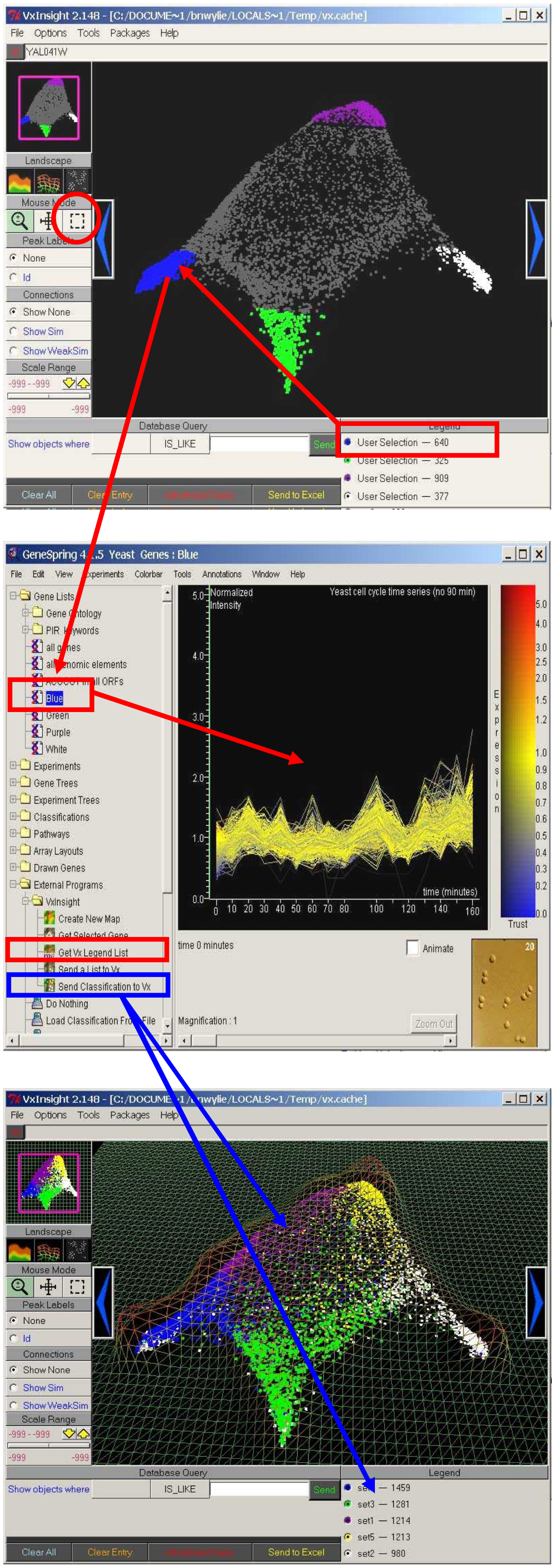

Example 1: A user creates a new map of all the genes. She than selects a particular cluster of interest in VxInsight and sends those gene back to GeneSpring and creates a new gene list. The gene list can now be graphed and manipulated. After deciding these are the genes she is really interested in, she can now select that list in GeneSpring and 'Create New Map' in VxInsight. Now a new map will be created with just those genes, and new features and clusters will become apparent in the subset. The user could repeat this process over and over.

©VisWave, LLC, 1997-2004

Example 2: A user creates a new map but is unsure of the meaning of the features and clusters displayed in the map. She has been working with a classification scheme in GeneSpring for a few weeks and would like to see that classification overlaid on the VxInsight map. She can simply select that classification in GeneSpring and click on the 'Send Classification to Vx' icon. The classification is now colored coded on the VxInsight Map (as seen in the figure on the previous page with a K-Means of 5 shown on the map). Now that she has some additional information, she now wants to see one group within the classification. She selects the classification and, within GeneSpring, creates lists out of that classification. Any one of these lists can now be separately displayed in the Vx map, and any one of the lists could be used to generate a new map.

Some of the other icons in GeneSpring include 'Get Selected Gene' and 'Open Map'. *Get Selected Gene*, retrieves one gene, the currently selected gene in VxInsight, and makes a list within GeneSpring. *Open Map* is an important time saver. When creating a new map out of tens of thousands of genes, the processing can take several minutes. After creating the map, it can be saved by selecting 'File...Save...' within VxInsight. Later on the user can simply click on the 'Open Map' icon within GeneSpring to quickly bring up the saved VxInsight map instead of recomputing it from scratch.

Loading your own Plugin into VxInsight

1) What do I need to create a plugin?

The plugin mechinism is currently based on tcl/tk. So your plugin has to be tcl based and your files are 'sourced' when activated.

2) How do I get my plugin to show up?

A 'plugins' directory is created within the VxInsight directory at installation. Simply create a new directory under plugins. That directory name will show up under the plugin menu. Inside the directory put a file called plugin.tcl. If you have additional files (tcl or other) that are part of the plugin they should be put in the same directory.

For example

```
plugins/
      genome/
            plugin.tcl
            foo.tcl
            datafile.txt
            etc...
```

This will result in just 'genome' being listed in the plugin menu.

3) How does my plugin get activated?

When the user selects your plugin name from the menu, the plugin.tcl file is 'sourced' and all tcl commands it that file are run. The plugin.tcl can 'source' other tcl files (path is relative).

4) How do I exchange information back and forth to VxInsight?

To exchange information the following command is available:

Vx_serviceRemoteRequest REQUEST_TYPE data

Request types currently available (see www.viswave.com for any updates)

MARK_OBJECTS:
- data = ids seperated by eoln
- This interaction highlights all ids in the data with the next available color and put the entry in the legend

GET_SELECTED:
- data = None
- This interaction returns the currently selected id

GET_EXPORT_LIST:
- data = None
- This interaction returns the currently selected legend lists

NEW_COORDS:
- data = coordinate filename
- This interaction tells VxInsight to import the specified

coordinate file

NEW_CACHE:

data = cache filename

This interaction tells VxInsight to import the specified cache file

OPEN_CACHE:

data = None

This interaction tells VxInsight to open a dialog where the user specifies the cache file

ORDINATE:

data = sim filename

This interaction tells VxInsight to ordinate the existing cache objects with the specified similarity file

5) Can I make additional menu entries in the Vx menubar?

Yes, the tk path of the menu bar is given by the following command

```
    Vx_getMenuBar
```

Example usage:

```
    set menu_path [Vx_getMenuBar]

    $menu_path add cascade -label "foo" -menu $menu_path.foo

    # foo command
    menu $menu_path.foo
    $menu_path.foo add command -label "MyCoolThing" -command
"my_tcl_command"
```

SUPPORT AND CONTACTS

As a licensed user of VxInsight you are entitled to 6 months support with your purchase. Additional support can be purchased from VisWave (see website for details and pricing).

VisWave is committed to selling their customers a solution, not just a piece of software. If you cannot read in your data please contact VisWave and we will guide you through the process or where possible write a module for VxImport that will help automate your process.

Please visit our website, it will contain up to date documentation and helpful information.
VisWave website: www.viswave.com

General Contact Information
Support Issues: support@viswave.com or call us at (505) 798-9304.
Licensing Issues: sales@viswave.com or call us at (505) 798-9304.

General Company Information
VisWave LLC
12042 Sundial NE
Albuquerque NM, 87122

Phone: (505) 798-9304
FAX: (505) 798-9304 *(this general line will 'sense' a fax and convert over)*
